# Cyclin F–EXO1 axis controls cell cycle–dependent execution of double-strand break repair

**DOI:** 10.1126/sciadv.ado0636

**Published:** 2024-08-09

**Authors:** Hongbin Yang, Shahd Fouad, Paul Smith, Eun Young Bae, Yu Ji, Xinhui Lan, Ava Van Ess, Francesca M. Buffa, Roman Fischer, Iolanda Vendrell, Benedikt M. Kessler, Vincenzo D’Angiolella

**Affiliations:** ^1^ MRC Weatherall Institute of Molecular Medicine, University of Oxford, John Radcliffe Hospital, Headington, Oxford OX3 9DS, UK.; ^2^ Edinburgh Cancer Research, CRUK Scotland Centre, Institute of Genetics and Cancer, University of Edinburgh, Crewe Road South, Edinburgh EH4 2XU, UK.; ^3^ Medical Science Division, University of Oxford, Oxford OX3 7DQ, UK.; ^4^ Department of Computing Sciences and the Bocconi Institute for Data Science and Analytics, Bocconi University, Milan, Italy.; ^5^ Target Discovery Institute, Centre for Medicines Discovery, Nuffield Department of Medicine, University of Oxford, Oxford OX3 7FZ, UK.; ^6^ Chinese Academy for Medical Sciences Oxford Institute, Nuffield Department of Medicine, University of Oxford, Roosevelt Drive, Oxford OX3 7FZ, UK.

## Abstract

Ubiquitination is a crucial posttranslational modification required for the proper repair of DNA double-strand breaks (DSBs) induced by ionizing radiation (IR). DSBs are mainly repaired through homologous recombination (HR) when template DNA is present and nonhomologous end joining (NHEJ) in its absence. In addition, microhomology-mediated end joining (MMEJ) and single-strand annealing (SSA) provide backup DSBs repair pathways. However, the mechanisms controlling their use remain poorly understood. By using a high-resolution CRISPR screen of the ubiquitin system after IR, we systematically uncover genes required for cell survival and elucidate a critical role of the E3 ubiquitin ligase SCF^cyclin F^ in cell cycle–dependent DSB repair. We show that SCF^cyclin F^–mediated EXO1 degradation prevents DNA end resection in mitosis, allowing MMEJ to take place. Moreover, we identify a conserved cyclin F recognition motif, distinct from the one used by other cyclins, with broad implications in cyclin specificity for cell cycle control.

## Teaser

E3 ubiquitin ligase cyclin F induces degradation of EXO1 in mitosis for correct deployment of microhomology-mediated end joining.

## INTRODUCTION

The maintenance of genomic integrity is a fundamental aspect of cellular homeostasis to allow cell survival. Among the different DNA lesions, double-strand breaks (DSBs) pose a threat to genome stability, as the improper repair of DSBs can lead to chromosomal rearrangements, oncogenic transformations, and cell death. To safeguard against these deleterious outcomes, cells have evolved an intricate and tightly regulated DNA damage response (DDR) network, including multiple DNA damage repair pathways. Homologous recombination (HR) and nonhomologous end joining (NHEJ) are the major DSB repair pathways in mammalian cells. NHEJ joins the DNA ends with minimal sequence homology in an error-prone manner, while HR uses the sister chromatid only available in late S/G_2_ cell cycle phases as a template to perform error-free repair. A key regulatory process in the DDR network is ubiquitination, a covalent and reversible posttranslational modification mediated by the ubiquitin-activating enzyme (E1), ubiquitin-conjugating enzyme (E2), and ubiquitin ligase (E3) cascade. Ubiquitination orchestrates the recruitment of DNA damage repair factors, signal transduction, and the temporal control of DNA repair pathways (*1*). Its role in coordinating HR and NHEJ during the cell cycle is controlled by several ubiquitination-dependent mechanisms (*2*, *3*). Besides these major repair pathways, microhomology-mediated end joining (MMEJ) and single-strand annealing (SSA) are considered backup mechanisms when HR and NHEJ are compromised (*4*). In contrast to the extensively studied HR and NHEJ, our knowledge of how MMEJ and SSA are executed and regulated remains scarce, except their error-prone nature and their characteristic dependence on DNA polymerase θ (POLQ) and RAD52, respectively (*5*). HR and SSA both require the exonuclease 1 (EXO1)– or DNA2-mediated long-range 5′ → 3′ nucleolytic degradation and the sequential exposure of a 3′ single-stranded DNA (ssDNA) overhang, a process known as long-range DNA end resection (*6*). EXO1- or DNA2-mediated DNA end resection can range from several hundred to a few thousand nucleotides long (*7*, *8*). Therefore, activities of these exonucleases need to be tightly regulated and precisely executed to avoid DNA breakage during damage repair, loss of genetic information, and unwanted recombination due to extensive homology exposure among random genomic regions. An example of such regulation is the posttranslational modification or direct inhibition of EXO1 upon DNA damage induction (*9*–*12*). Long-range resected DNA is known to be a poor substrate for NHEJ and MMEJ machineries, indicating a possible role of DNA end resection dictating DSB repair pathway choice. To date, various mechanisms have been reported to direct pathway choice between NHEJ and HR (*13*, *14*). However, the role of ubiquitination in regulating resection and alternative DSB pathway choice during cell cycle is understudied with major mechanisms remaining unknown.

Starting from a focused CRISPR screen to systematically discover E1s, E2s, and E3s that are required for cell survival after ionizing radiation (IR), we identify a crucial axis for cell cycle–dependent regulation of DSBs executed by the *CCNF* gene. Its encoded protein, cyclin F, is a substrate receptor of the SKP1-CUL1-F-box (SCF) E3 family known to degrade several substrates involved in cell cycle and in DNA damage repair (*15*–*18*). In this study, we observe that cyclin F degrades EXO1 in the G_2_/M cell cycle phase to allow the correct execution of MMEJ. Disruption of this axis either by *CCNF* knockout or by expressing a nondegradable R842A EXO1 mutant promotes hyper-resection and chromosome aberrations, increasing IR-induced cell death. Our study unravels an unknown mechanism to regulate DSB backup repair pathways during the cell cycle. The findings reported find related alterations in cancers treated with IR like glioblastomas, opening exciting prospects for developing more targeted therapeutic strategies.

## RESULTS

### CRISPR screen of the ubiquitin system identifies genes required for cell survival after IR

Radiotherapy by IR is used extensively to treat cancer in both palliative and curative contexts. For some tumor types, such as glioblastoma multiforme (GBM), radiotherapy remains the major therapeutic strategy besides surgery due to the scarcity of chemotherapy options. However, patients still suffer from dismal prognosis due to GBM cells’ fast development of radioresistance. It is, therefore, imperative to identify the major pathways conferring IR resistance to develop strategies to resensitize cancer cells to radiation. To this end, we decided to perform an unbiased CRISPR screen on genes involved in ubiquitin conjugation for the identification of radioprotective genes in GBM cells. To perform this screen in a radioresistant cell line, we tested the IR sensitivity of a panel of seven glioblastoma-derived cell lines via colony formation assay following IR (fig. S1A). Among the tested cell lines, LN229 was found to be the most radioresistant, providing an ideal model for dropout screens, a screen to identify genes whose depletion promotes radiosensitivity.

A clonal population of LN229 was then generated using lentiviral expressing system to reach a high level of Cas9 protein expression (fig. S1B). To minimize variations caused by Cas9 expression and clonal effects, we isolated several clones and selected the one that showed no differences in cell proliferation and radiosensitivity when compared to the LN229 parental cells (fig. S1C).

**Fig. 1.**
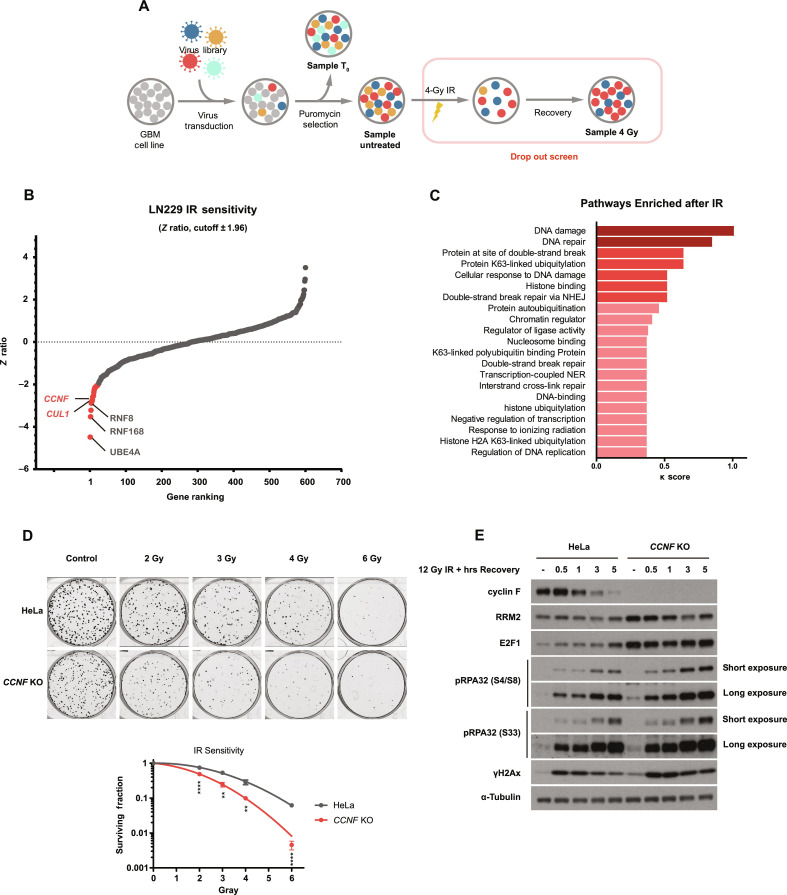
CRISPR screen of the ubiquitin system identified genes required for cell survival after IR. (**A**) Schematic representation of the CRISPR screen. Two days after transducing Cas9-expressing LN229 with the sgRNA library, sample T_0_ was collected as a reference sample of sgRNA expression. Cells were then selected with puromycin for 7 days before ionizing radiation (IR) treatment. Fourteen days after IR (4 Gy), cells were collected for genomic DNA extraction and PCR amplification of the sgRNA sequences. PCR product was sent for next-generation sequencing so that change in relative abundance of each sgRNA before and after IR could be assessed. (**B**) Identification of genes affecting IR sensitivity in LN229 by *z* ratio. Positive controls identified are highlighted in gray two hits from the screen are highlighted in red. See table S2 for the full list of genes. (**C**) Over-representation analysis (ORA) of statistically significant hits identified from the screen (absolute value of *z* ratio > 1.96). Pathways colored in dark red are highly confident (κ > 1). Pathways in red are confident (κ > 0.5). Pathways in pink are potential pathways (κ < 0.5). (**D**) Colony formation assay in *CCNF* K/O’s cells. HeLa parental cells or HeLa *CCNF* K/O cells were seeded for colony formation assay and challenged with the indicated dose of IR. Seven days after IR, cells were stained with crystal violet and counted. Error bars represent SDs of three biological replicates. Two-tailed unpaired *t* test was performed as statistical analysis. **P ≤ 0.01; ***P ≤ 0.001; ****P ≤ 0.0001. (**E**) Immunoblotting after IR treatment and recovery as indicated. DNA damage markers detected: pRPA32 S4/8 (ssDNA at DSB), pRPA32 S33 (single-strand breaks), and γH2Ax (DNA DSBs).

A commercially available library focused on the ubiquitin system was used in the screen. The library covers five ubiquitin-activating enzymes (E1s, including E1s for ubiquitin-like molecules), 35 ubiquitin-conjugating enzymes (E2s), 531 established or putative ubiquitin ligases (E3s), 31 core essential genes as positive controls, and 100 nontargeting controls. Each gene was targeted with 10 single guide RNA (sgRNA) guides and we choose a fold representation of ~750 for improved resolution of the screen (illustrated in Fig. 1A). Cells were treated with 4-gray (Gy) IR in a single dose, which was previously found to kill around 50% of LN229 cells via colony formation assays (fig. S1A and S1C).

The replicates of the screen showed significant correlation and clustering in the principal components analysis (fig. S1, D and S1E). In addition, we calculated the effect size for all guides using Cohen D (fig. S1F) (*19*). The score indicated a large effect size highlighting the statistical power of the screen.

We first analyzed the results in untreated cells, comparing cells harvested immediately after library transduction (sample T_0_) and cells that survived the puromycin selection (sample untreated). We identified positive control genes (core essential genes) provided in the library with a good precision recall (15 of 24) (fig. S1G and table S1). These results indicated the cells underwent selection through the effect exerted by the individual sgRNAs.

To identify the genes required for survival after IR, we compared cells treated with 4-Gy IR to the untreated cells. A *z* ratio score was plotted using CRISPR AnalyzeR (*20*) and presented in Fig. 1B (full *z* ratio ranking is available in table S2). Over-representation analysis (ORA) was performed on the statistically significant genes that conferred either IR sensitivity or IR resistance (with *z* ratio > 1.96 or < −1.96). A significant enrichment was detected for genes participating in DNA repair and DNA damage, as expected (Fig. 1C).

It is worth noting that we identified *UBE4A*, *RNF168*, and *RNF8* as top hits. These genes have crucial roles in mediating DDR and repair (*21*–*23*), indicating that the screen could reveal hitherto uncharacterized players in DDR and repair. We also identified *UBA3*, the E1 for conjugating the ubiquitin-like protein NEDD8, supporting the idea that neddylation is required in DDR. The neddylation inhibitor MLN4924 is a potent radiosensitizer but with unknown mechanism (*24*–*26*). In addition to these well-established DNA damage regulators, we also identified CUL1, the central component protein of SCF E3 complexes, and several F-box proteins, which function as the substrate receptors for SCFs.

Among the SCF genes identified from the screen, cyclin F ranked the highest among all F-box adaptors. To test the IR sensitivity induced by loss of cyclin F, we performed a colony formation assay in HeLa cells where the *CCNF* gene was knocked out using CRISPR. *CCNF* knockout (*CCNF* K/O) sensitized HeLa cells to IR at all doses tested (Fig. 1D).

To have an overview on the DDR pathways affected by *CCNF* K/O, we checked several DDR markers as indicated below. We observed up-regulated steady state levels of the previously reported cyclin F substrates, RRM2 (*15*) and E2F1 (*17*). In addition, compared to the control cells, the delivery of IR to *CCNF* K/O cells induced increase of RPA32 phosphorylation at Ser^4^/Ser^8^ (a marker of ssDNA at DSB repair sites upon Ataxia telangiectasia mutated and DNA-PK activation), Ser^33^ (marker of ssDNA upon Ataxia telangiectasia related activation) and γH2Ax levels at steady-state level and after IR (Fig. 1E). These results indicate that loss of cyclin F induces basal level DNA damage and prevents or delay the repair of damaged DNA after IR treatment.

### Cyclin F interacts with and ubiquitinates EXO1 during G_2_/M and after IR

**Fig. 2.**
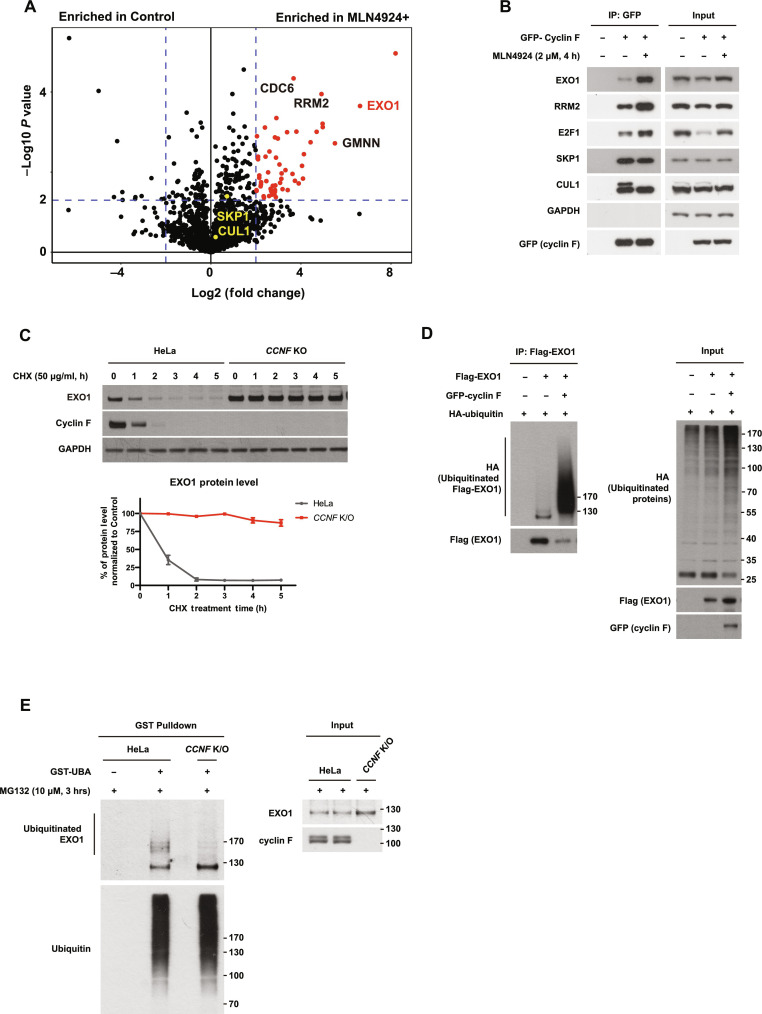
Cyclin F interacts and ubiquitinates EXO1. (**A**) Volcano plot representing MS analysis of differential TurboID–cyclin F interacting partners in the presence versus absence of MLN4924. Proteins were isolated with streptavidin after labeling for 1 hour with biotin. (**B**) Immunoblotting after IP of GFP–cyclin F in HEK293T treated with MLN4924 as indicated. Input samples before IP are presented on the right. (**C**) Immunoblotting after treating cells with cycloheximide (CHX) for the indicated time (h = hour) (top). Relative quantification of EXO1 protein levels in HeLa parental cells or *CCNF* K/O cells after normalization with EXO1 levels at T0 for each cell line (bottom). Error bars: SDs of three biological replicates. (**D**) Immunoblotting after expression of GFP–cyclin F, Flag-EXO1, and HA-ubiquitin in HEK293T. Flag-EXO1 is isolated via Flag agarose beads pulldown before immunoblotting. Input samples before IP are presented on the right. (**E**) Immunoblotting after isolation of endogenous ubiquitinated proteins using recombinant GST-tagged UBA domain of UBQLN1 protein. Input samples before IP are on the right.

To understand how cyclin F regulates IR sensitivity, DDR, and repair, we established a cell line expressing cyclin F fused to TurboID (an engineered biotin ligase that conjugates biotin to proteins) to identify the proteins interacting with cyclin F (fig. S2A). This approach, which exploits a nonspecific biotin ligase, has been used extensively to identify interacting partners of cellular proteins (*27*) with improvements made to enhance specificity/efficiency (*28*). In our approach, we compared untreated cells to cells treated with MLN4924 to generate a comprehensive view of potential cyclin F substrates (Fig. 2A and table S3). MLN4924 helps to enrich cyclin F–substrate interactions by inhibiting the action of ligating ubiquitin onto substrates and therefore the subsequent dissociation of cyclin F from its substrates. As shown in Fig. 2A and table S3, several hits are significantly enriched in the MLN4924-treated condition, with EXO1 identified as one of the top hits.

Interaction between cyclin F and EXO1 was validated (Fig. 2B). MLN4924 promoted cyclin F interaction with the known substrates E2F1, RRM2, and with EXO1. Interaction between cyclin F and the SCF components CUL1 and SKP1 remained intact, while Cul1 neddylation was abolished by MLN4924 (Fig. 2B).

To test whether EXO1 is an SCF^cyclin F^ substrate targeted for degradation, we measured the half-life of EXO1 after challenging cells with cycloheximide (CHX) to block translation of proteins. As shown in Fig. 2C, the half-life of EXO1 is less than 1 hour in parental cells but increases to over 5 hours in *CCNF* K/O cells (Fig. 2C). Similar results were obtained with a small interfering RNA targeting cyclin F in LN229 cells (fig. S2B). Conversely, overexpressed cyclin F was found to reduce EXO1 protein levels in both LN229 and human embryonic kidney (HEK) 293T. The observed reduction of EXO1 is rescued by the proteasome inhibitor MG132 (fig. S2C), demonstrating that the reduced levels of EXO1 are due to proteasomal degradation of EXO1 induced by cyclin F elevated expression.

To establish a direct role of cyclin F in ubiquitinating and degrading EXO1, we used two methods to detect EXO1 ubiquitination. We isolated EXO1 protein after cyclin F elevated expression, observing a substantial increase laddering of bands corresponding to EXO1 ubiquitination (Fig. 2D). The nonphysiological expression of E3 ligases and ubiquitin itself can lead to artifactual ubiquitination events; thus, we further detected EXO1 ubiquitination at endogenous level. After unbiasedly enriching all ubiquitinated proteins from parental HeLa cells using the recombinant ubiquitin-associated domain (UBA domain) of the UBQLN1 protein in a ubiquitin binding entity (UBE) pulldown assay (*29*), we detected endogenous polyubiquitinated EXO1 in HeLa control cells (Fig. 2E). The polyubiquitinated smear signal was completely removed in *CCNF* K/O cells, suggesting cyclin F as a major E3 responsible for the ubiquitination of endogenous EXO1 (Fig. 2E).

To further elucidate the ubiquitin chains specificity mediated by SCF^cyclin F^ on EXO1, we used a K48R ubiquitin mutant (K48R). As shown in fig. S2D, the K48R mutation in ubiquitin completely abrogated both basal-level EXO1 ubiquitination and cyclin F overexpression-induced EXO1 ubiquitination, demonstrating that EXO1 ubiquitination is mainly through K48 linkages.

We observed reduced levels of EXO1 3 hours after IR treatment in accordance with findings by Tomimatsu *et al.* (*9*) The reduction of EXO1 levels is also cyclin F–dependent because in *CCNF* K/O cells, we could detect high levels of EXO1 after IR (fig. S3A). We measured interaction between EXO1 and cyclin F at different time points after IR treatment and detected an increase in the interaction between cyclin F and EXO1 over time, suggesting that cyclin F also promotes EXO1 degradation after IR (fig. S3B). We cannot exclude that the degradation of EXO1 in these conditions is triggered by cells moving from S phase to mitosis after IR, but the interaction between cyclin F and RRM2 was reduced in the same experiments, suggesting the presence of additional modes of EXO1 regulation after DNA damage. The cyclin F–EXO1 interaction was also promoted by treatment with camptothecin, a topoisomerase I inhibitor known to arrest cells in S phase (fig. S3C). Furthermore, endogenous ubiquitination of EXO1 was increased after treating cells with IR as detected by TUBE assay. The ubiquitination ladder was dependent on cyclin F as it disappeared upon cyclin F depletion (fig. S3D).

Together, our findings demonstrate that cyclin F is the main E3 ligase targeting EXO1 for K48-linked polyubiquitination both in mitosis and after DNA damage.

### EXO1 accumulation mediates increased sensitivity to IR upon cyclin F depletion

We have established that EXO1 is targeted for ubiquitination and degradation by cyclin F in G_2_/M phases of the cell cycle; however, the functional significance of this regulation remains unclear. Since EXO1 is one of the key exonucleases in DNA end resection after DSBs and participates in DNA damage repair, we speculated that it could be the main substrate whose accumulation leads to IR hypersensitivity in *CCNF* K/O cells.

**Fig. 3.**
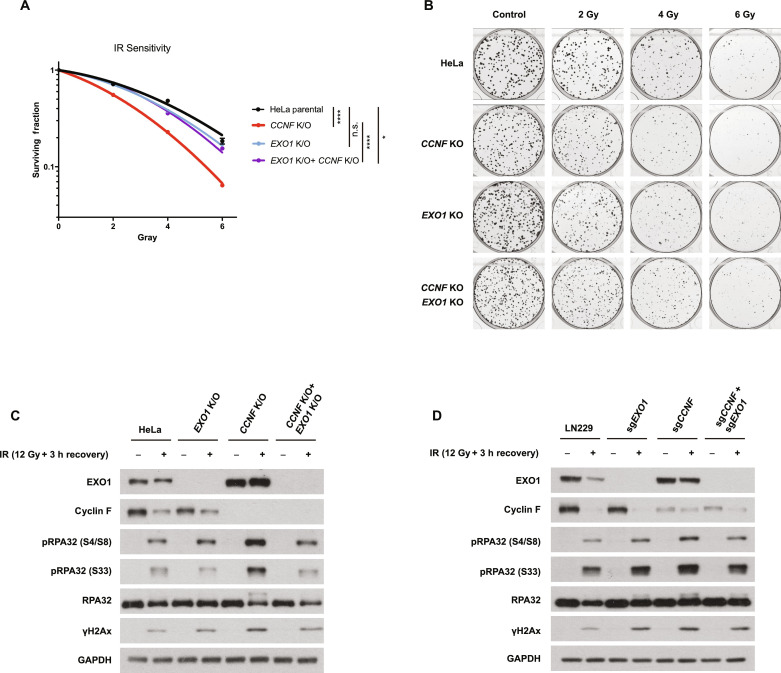
EXO1 accumulation mediates increased sensitivity to IR upon cyclin F depletion. (**A**) HeLa parental cells, HeLa *CCNF* K/O, HeLa *EXO1* K/O, and HeLa *CCNF* K/O *EXO1* K/O as indicated were seeded for colony formation assay and challenged with the indicated doses of IR. Seven days after IR, cells were stained with crystal violet and counted. Error bars represent SDs of three biological replicates. Two-tailed unpaired *t* test was performed as statistical analysis. **P ≤ 0.01; ***P ≤ 0.001; ****P ≤ 0.0001. (**B**) Colony formation assay representative image of (A). (**C**). Immunoblotting in HeLa cells after IR treatment and recovery as indicated. DNA damage markers detected: pRPA32 S4/8 (ssDNA at DSB), pRPA32 S33 (single strand breaks), and γH2Ax (DNA DSBs). (**D**) Immunoblotting in LN229 cells after IR treatment and recovery as indicated. DNA damage markers detected as in (C). LN229 cells were transiently transfected with Cas9 protein and sgRNA, as indicated, 4 days before IR treatment.

To test our hypothesis, we generated *EXO1* knockout (*EXO1* K/O) cells within a *CCNF* K/O background. In total, we generated four cell lines: HeLa, HeLa *EXO1* K/O, HeLa *CCNF* K/O, and HeLa *CCNF* K/O *EXO1* K/O (double K/O for cyclin F and EXO1) and assessed their sensitivity to IR. *CCNF* K/O showed increased sensitivity (Fig. 1D), while EXO1 removal by K/O did not have a notable impact on IR sensitivity (Fig. 3, A and B). In accordance with our hypothesis, the depletion of *EXO1* in a *CCNF* K/O background fully rescued IR sensitivity observed in *CCNF* K/O, supporting the idea that EXO1 accumulation upon cyclin F loss is responsible for the IR hypersensitivity detected in *CCNF* K/O cells (Fig. 3, A and B). Moreover, DNA damage markers after IR further endorsed this observation at molecular level (Fig. 3C) where elevated levels of RPA32 phosphorylation at S33 and S4/8 caused by *CCNF* K/O were fully rescued by *EXO1* depletion in the cell line where both EXO1 and cyclin F were depleted. Similar results were obtained in a different cell line (LN229) using sgRNAs targeting *CCNF* and *EXO1* (Fig. 3D). Together, these data demonstrate that the IR hypersensitivity observed upon cyclin F depletion is due to EXO1 accumulation.

### EXO1 T824 phosphorylation by CDK1/cyclin A is required for the interaction with cyclin F and subsequent ubiquitination

The mechanism of EXO1 recognition by cyclin F is not known, so we mapped the domains mediating the interactions on both proteins. We and others have previously shown that cyclin F uses the cyclin domain to recruit substrates (*16*, *30*). To establish that the cyclin domain is required for EXO1 recruitment and ubiquitination, we tested EXO1 interaction with two cyclin F mutants previously reported (*15*, *16*), ΔF (with L45A and P46A mutation in cyclin F’s F-box domain which compromises SKP1-CUL1 interaction) and ΔC (with M309A mutation, which disrupts cyclin F’s cyclin domain), respectively. Both wild-type cyclin F (depicted as WT) and ΔF were able to pulldown endogenous EXO1, while the ΔC mutant was not (fig. S4A). Ubiquitination experiments conducted with the same cyclin F mutants demonstrated a remarkable reduction in EXO1 ubiquitination in the ΔF condition and a complete loss of ubiquitination in the ΔC condition (fig. S4B). Together, these results demonstrate that cyclin F interacts with EXO1 through its cyclin domain.

**Fig. 4.**
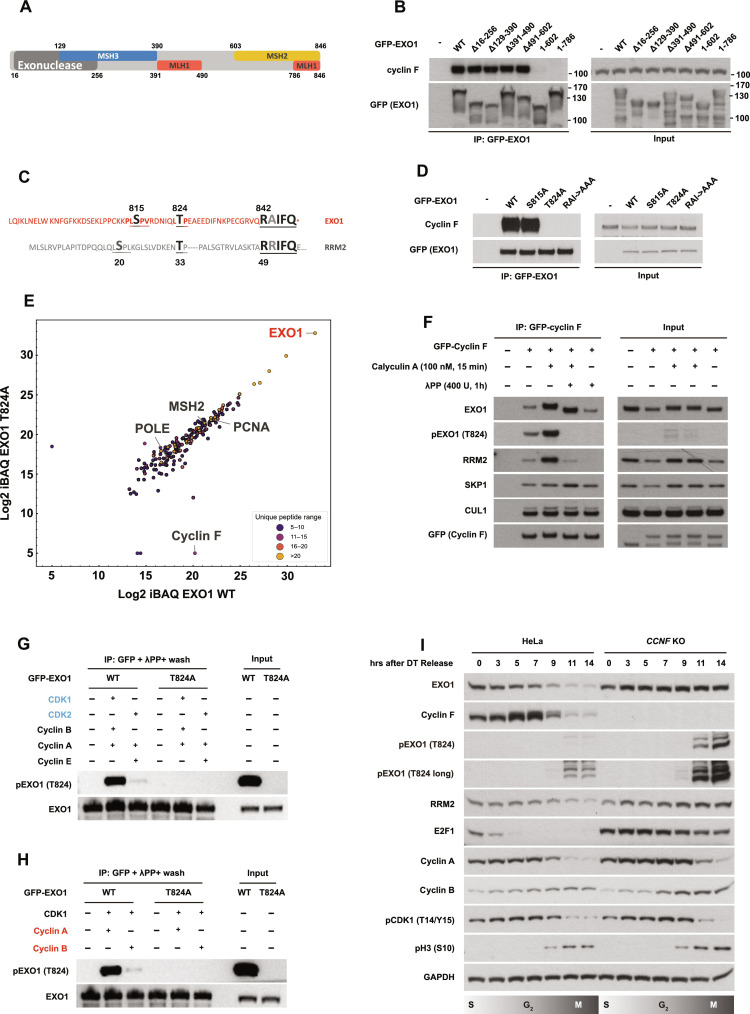
EXO1 T824 phosphorylation by CDK1/cyclin A is required for the interaction with cyclin F and subsequent ubiquitination. (**A**) Schematic representation of EXO1 depicting the domains and known interacting partners. (**B**) Immunoblotting after IP of GFP-EXO1 WT and the indicated EXO1 fragments. Input samples before IP are presented on the right. (**C**) The last 60 amino acids of EXO1 C terminus are highlighted and aligned with cyclin F interaction motif on RRM2. Amino acids conserved in both EXO1 and RRM2 are enlarged and labeled in black/gray. (**D**) Immunoblotting after IP of GFP-EXO1 WT and the indicated GFP-EXO1 mutants (S815A, T824A, and RAI/842-844/AAA). Input samples before IP are presented on the right. (**E**) Identification of differential interactors by MS after IP of Flag-EXO1 WT versus Flag-EXO1 T824A. Unique peptide ranges are labeled with the indicated colors. (**F**) Immunoblotting after IP of GFP–cyclin F plus treatment with calyculin A or λPP as indicated. λPP treatment was conducted on beads after IP. Input samples before IP are presented on the right. (**G**) Immunoblotting of in vitro phosphorylation assay using GFP-EXO1 WT or GFP-EXO1 T824A purified from HEK293T cells as substrates and the recombinant cyclin E/CDK2, cyclin A/CDK2, and cyclin A-B/CDK1 as indicated. Isolated GFP-EXO1 WT or GFP-EXO1 T824 mutant were dephosphorylated by λPP on beads before being used for in vitro phosphorylation. (**H**) Immunoblotting of in vitro phosphorylation assay using GFP-EXO1 WT or GFP-EXO1 T824A purified from HEK293T cells as substrates and recombinant cyclin A/CDK1 or cyclin B/CDK1 as indicated. (**I**) Immunoblotting of cell cycle synchronized HeLa cells via double thymidine (DT) block release.

Similarly, we mapped cyclin F interaction site(s) on EXO1. To this end, EXO1 fragments encompassing the exonuclease domain (amino acid residues from 16 to 256), MSH3 interaction domain (129 to 390), MLH1 interaction domain part 1 (391 to 490), MSH2 interaction domain (603-end), and MLH1 interaction domain part 2 (786-end) were generated and tested for their interactions with endogenous cyclin F (domains are depicted in Fig. 4A). As shown in Fig. 4B, only the C-terminal truncation on EXO1 disrupted its interaction with cyclin F, indicating the requirement of the last 60–amino acid residues on EXO1 for cyclin F interaction. Alignment of the last 60–amino acid residues in EXO1 protein with the previously identified RRM2 degron revealed notable similarity in the two proteins: A T824 residue in EXO1 closely resembles the T33 in RRM2, which was previously identified as the priming phosphorylation site in initiating cyclin F binding, and an RxIFQ motif spanning from 842 to 846 is equivalent to the residues 49 to 53 in RRM2 (Fig. 4C), including an RxI motif previously identified as essential for cyclin F interaction (*15*). Therefore, we tested whether the conserved residues are required for cyclin F–EXO1 interaction.

As presented in Fig. 4D, both an EXO1 mutant lacking the T824 and an EXO1 lacking the RxI at position 842-845 RxI were unable to recruit cyclin F, highlighting a similar recognition mechanism to RRM2. Unbiased analysis of interacting proteins with immunoprecipitated EXO1 WT and EXO1 T824A identified cyclin F as the major differential protein (Fig. 4E and table S4). As expected, EXO1 T824A mutant could not be ubiquitinated by cyclin F (fig. S4C). On the contrary, when the serine-815 residue in EXO1 was mutated to alanine, there was no detectable difference in EXO1–cyclin F interaction and EXO1 ubiquitination (Fig. 4D and fig. S4C). These data show that EXO1–cyclin F interaction and SCF^cyclin F^–mediated ubiquitination are both dependent on the presence of T824 in EXO1.

To establish whether T824 is phosphorylated in vivo, we raised antibodies specific to phosphorylated T824 on EXO1 and validated them after immunoprecipitation of EXO1 (fig. S4D) and dot blot with purified peptides (fig. S4E). One of the two affinity-purified polyclonal antibodies was found to be highly specific to phosphorylated T824 EXO1 (fig. S4E). To establish that the antibody specifically recognizes the phosphorylated form of EXO1 in cell extracts, we treated cells with calyculin A, a potent inhibitor of both protein phosphatase 1 (PP1) and protein phosphatase 2A (PP2A), to elevate global phosphorylation in cells. In addition, we also treated immunoprecipitated proteins with λ phosphatase (λPP), a Mn^2+^-dependent protein phosphatase with activity toward phosphorylated serine, threonine, and tyrosine. Treatment with calyculin A was found to elevate the EXO1 pT824 signal, while treatment with λPP completely removed it (Fig. 4F), indicating that EXO1 is phosphorylated on T824. The interaction data obtained in these experiments described above supported the idea that phosphorylation was required for interaction but not essential. Calyculin A treatment promoted cyclin F’s interaction with RRM2 and with EXO1. λPP treatment after calycuclin A reduced cyclin F–RRM2 interaction back to the untreated levels and had no notable effect on the interaction between cyclin F and EXO1 (Fig. 4F). Together, these data are in accordance with a model where T824 phosphorylation is required to initiate cyclin F–EXO1 interaction but is dispensable for the maintenance of the interaction.

To identify the kinase responsible for T824 phosphorylation, we used the site-specific phosphorylation antibody to detect EXO1 T824 phosphorylation after challenging cells with a panel of kinase inhibitors involved in the cell cycle and DDRs. While several inhibitors affected cyclin F levels and its interaction with EXO1, only RO-3306 (CDK1 inhibitor) and K03861 (CDK2 inhibitor) induced a remarkable reduction in both cyclin F–EXO1 interaction and EXO1 T824 phosphorylation (fig. S4F). BI6727 (PLK1 inhibitor) and CKIIi VIII (casein kinase II inhibitor) also drastically down-regulated cyclin F–EXO1 interaction but did not impact on T824 phosphorylation (*31*) (fig. S4F). Thus, the kinases phosphorylating EXO1 on T824 are CDKs in line with the degradation of EXO1 at G_2_/M when CDK activity peaks.

To further define the specific cyclin/CDK partner(s) responsible for the T824 phosphorylation, we implemented an in vitro phosphorylation assay using CDK2/cyclin E, CDK2/cyclin A, CDK1/cyclin A, and CDK1/cyclin B. A substantial increase in EXO1 T824 phosphorylation was detected only upon the addition of CDK1 into the reaction but not CDK2, regardless of the types of the cyclin proteins (Fig. 4G). Moreover, CDK1/cyclin A and CDK1/cyclin B were tested for their capability in phosphorylating EXO1 at T824. Only CDK1/cyclin A induced EXO1 T824 phosphorylation (Fig. 4H).

Cyclin F oscillates during the cell cycle peaking in G_2_ phase; therefore, cyclin F substrates are expected to be mainly degraded in the G_2_/M cell cycle phases (*15*–*17*). To test this, cell cycle synchronization was performed using double thymidine block in control HeLa cells and *CCNF* K/O cells to assess EXO1 protein levels. As shown in Fig. 4I, EXO1 protein level started to decrease 7 hours after cells were released into thymidine-free media, the exact time when cyclin F level peaks in G_2_. EXO1 is almost entirely depleted at the 14-hour time point when cells enter mitosis (evidenced by two markers of CDK1 activation, phosphorylation of histone H3 on serine-10 and loss of phosphorylated CDK1 on threonine-14/tyrosine-15). EXO1 down-regulation in G_2_/M, observed in control cells, was prevented by *CCNF* K/O (Fig. 4I). Phosphorylation of EXO1 on T824 accumulated specifically in mitotic cells but drastically increased upon cyclin F K/O. Together, these results demonstrate that cyclin A/CDK1 phosphorylates EXO1 at T824 to prime EXO1 degradation at the G_2_/M transition.

### Cyclin F interaction with EXO1 define a specific bivalent recognition domain (F-deg)

**Fig. 5.**
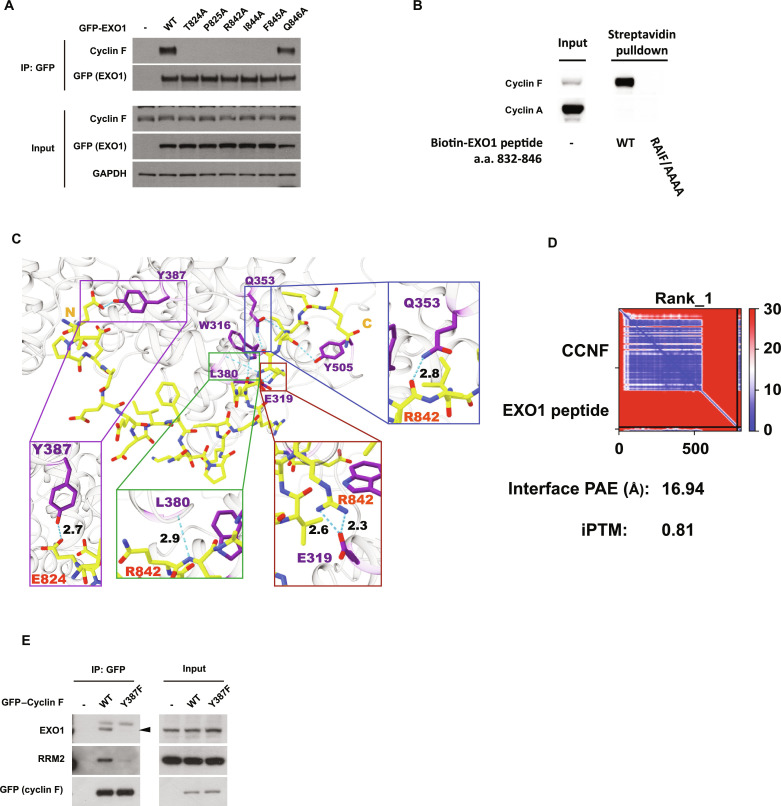
Cyclin F interaction with EXO1 define a specific bivalent recognition domain (F-deg). (**A**) Immunoblotting after IP of GFP-EXO1 WT, GFP-EXO1 T824A, GFP-EXO1 P825A, GFP-EXO1 R842A, GFP-EXO1 I844A, GFP-EXO1 F845A, and GFP-EXO1 Q846A mutants as indicated. Input samples before IP are presented at the bottom. (**B**) Immunoblotting after pulldown with streptavidin from cell extracts using as bait a biotinylated peptide encompassing EXO1 832-846 or a biotinylated peptide EXO1 832-846 where the RxIF residues were changed to alanine. (**C**) Docking of EXO1 peptide to cyclin F protein using COSMIC2. The peptide sequence from L823 to Q846 of EXO1 was used for the prediction. To mimic the phosphorylated status of EXO1, T824 was substituted to an E before docking. Cyclin F structure used in the docking is a predicted structure by AlphaFold Protein Structure Database. The iptm + ptm value is 0.7984765224933627. (**D**) Predicted aligned error (PAE) value plot of the prediction represented in (C). (**E**) Immunoblotting after IP of GFP–cyclin F WT or GFP–cyclin F Y387F mutant as indicated. Input samples before IP are presented on the right.

The alignment of the shared recognition sequence in RRM2 and EXO1 revealed additional similarities, which extended the potential recognition site from RxI to RXIFQ (Fig. 4C). Thus, we decided to test the essentiality of each of the amino acid residues in the RxIFQ in both RRM2 and EXO1. In both cyclin F substrates, the mutation of R, I, and F to A prevented substrate interaction with cyclin F, while substitution of Q to A did not impact on cyclin F’s interaction with both EXO1 and RRM2 (Fig. 5A and fig. S5A). To establish that the RxIF was the essential motif required for the interaction with cyclin F, we synthesized peptides containing the last 15 amino acids of EXO1 with the RxIF motif but lacking the T824 residue. This peptide was sufficient to retrieve cyclin F from cell lysates, while mutations in the peptide substituting RxIF residues to A completely abolished its interaction with cyclin F (Fig. 5B). The latter experiment shows that the RxIF is sufficient for interaction between cyclin F and EXO1, further supporting the idea that phosphorylation of EXO1 is a priming mechanism to initiate the interaction with the RxIF.

To gain insights into the priming mechanism of phosphorylation within EXO1, we used Alphafold multimer to dock cyclin F on an EXO1 peptide spanning the phosphorylation site and the RxIF motif (Fig. 5C). To obtain confidence of our prediction, we calculated the mean interface–predicted aligned error values for the three top-ranked predictions, as previously done (Fig. 5D and fig. S5D) (*32*). The top-ranking model highlights hydrogen bonds between EXO1’s R842 residue and cyclin F’s E319 residue, which closely resembles the interaction between cyclin A and the CDK inhibitor p27 [R30 residue in p27 and E220 residue in cyclin A (*33*)]. In addition, the same model predicts a potential hydrogen bond forming between EXO1’s T824 (mutated to E824 to mimic phosphorylated residue) and cyclin F’s Y387, indicating a potential second valence mediating the cyclin F–EXO1 interaction. To test the validity of this prediction, we mutated Y387 to phenylalanine in cyclin F and tested interaction with EXO1. Cyclin F Y387P was unable to interact with EXO1, in support of a multivalent mode of interaction between cyclin F and its substrates (Fig. 5E). The identification of the RxIF motif and a phosphorylation site in proximity facilitated a homology-based search within the human proteome for other potential cyclin F substrates. The search retrieved 34 matches (fig. S5B), including several well-established cyclin F substrates and interactors identified by liquid chromatography–mass spectrometry (LC/MS) by us and other groups (*34*–*36*). Among the potential substrates, NEK9 was successfully validated as a cyclin F interactor (fig. S5C), establishing the F-deg motif as a bona fide recognition site for cyclin F.

The results above indicate a potential bivalent recognition site for cyclin F with important implications for cyclin specificity during cell cycle progression. The described bivalent recognition site may prevent uncontrolled degradation of CDK/cyclin substrates by cyclin F at the G_2_/M transition.

### EXO1 R842A mutation phenocopies *CCNF* K/O

**Fig. 6.**
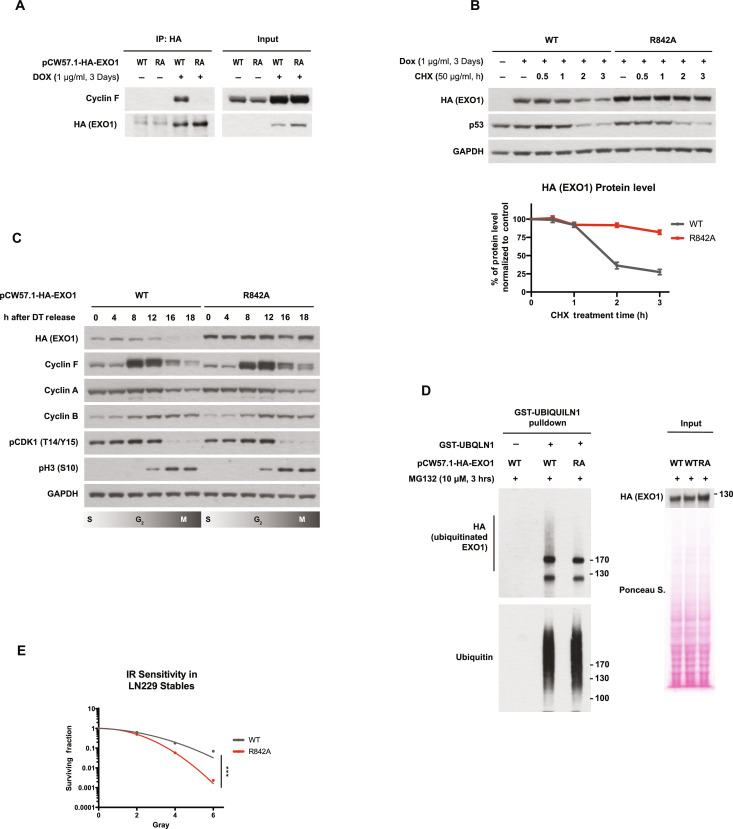
EXO1 R842A mutation phenocopies *CCNF* K/O. (**A**) Immunoblotting after IP of stably integrated HA-EXO1 WT or HA-EXO1 R842A in LN229 using a doxycycline-inducible promoter. Cells were treated with doxycycline (1 μg/ml) for 3 days to induce expression. Input samples before IP are on the right. (**B**) Immunoblotting after treating cells described in (A) with CHX for the indicated time (top). Relative quantification of EXO1 protein levels in HA-EXO1 WT or HA-EXO1 R842A after normalization with EXO1 levels at T_0_ for each cell line (bottom). Error bars: SDs of three biological replicates. (**C**) Immunoblotting of cell cycle synchronized cells described in (A) via DT block release. (**D**) Immunoblotting after isolation of endogenous ubiquitinated proteins using recombinant GST-tagged UBA domain of UBQLN1 protein of cells indicated in (A). Input samples before IP are on the right. (**E**) LN229 cells expressing HA-EXO1 WT or HA-EXO1 R842A were seeded for colony formation assay and challenged with the indicated dose of IR. Seven days after IR, cells were stained with crystal violet and counted. Error bars represent SDs of three biological replicates. Two-tailed unpaired *t* test was performed as statistical analysis. Two-tailed unpaired *t* test. **P ≤ 0.01; ***P ≤ 0.001; ****P ≤ 0.0001.

As shown in Fig. 3A, EXO1 is the major mediator of IR sensitivity upon *CCNF* loss (Fig. 3, C and D); therefore, it is reasonable to hypothesize that EXO1 stabilization phenocopies *CCNF* loss in regard to IR sensitivity. To test this hypothesis in a refined system, we generated cell lines that, upon doxycycline induction, express hemagglutinin (HA)–tagged EXO1 WT or HA-tagged EXO1 R842A mutant (which, according to previous data, abrogates cyclin F–EXO1 interaction and EXO1 ubiquitination). As validation in Fig. 6A shows, both WT and R842A mutant EXO1 were successfully expressed upon doxycycline induction, with R842A demonstrating a higher protein level in the input (Fig. 6A). EXO1 R842A interaction with cyclin F was abolished compared to that of the WT (Fig. 6A). Moreover, the half-life of EXO1 R842A was longer than EXO1 WT, proving that EXO1 R842A mutant is resistant to cyclin F–mediated degradation (Fig. 6B). In a cell cycle synchronization experiment, we also observed that HA-EXO1 WT was degraded in G_2_/M when cyclin F level peaks, while HA-EXO1 R842A was not (Fig. 6C), confirming that the cell cycle regulation of EXO1 is directly mediated by cyclin F through the interaction with the F-deg. Last, enrichment of the endogenous ubiquitinated proteins using TUBE pulldown assays confirmed that EXO1 WT can be ubiquitinated while EXO1 R842A cannot (Fig. 6D), showing a major role for cyclin F in EXO1 ubiquitination.

Given that IR sensitivity in cyclin F–depleted cells can be fully rescued by concomitant EXO1 depletion (Fig. 3A), EXO1 R842A mutant is expected to recapitulate the same radiosensitization phenotype observed in *CCNF* K/O cells (as shown in Fig. 1D). Colony formation assay in Fig. 6E demonstrated that cells expressing the EXO1 R842A mutation are significantly more sensitive to IR compared to cells expressing EXO1 WT. Moreover, steady-state γH2Ax level was also found to be increased in R842A mutant (fig. S6C).

A point mutation at R842 was identified in a glioblastoma patient within the Cancer Genome Atlas Program (fig. S6A). Although the arginine residue was mutated to an isoleucine in this case, the R842I mutation disrupts cyclin F–EXO1 interaction (fig. S6B). It is possible to speculate that the good prognosis of this patient at 6 months might be due to an exceptional response to radiotherapy as observed in other cases bearing alterations in other DDR genes (*37*). However, the patient was not followed beyond 6 months, making the prediction of good prognosis difficult at this stage. Nonetheless, our observation suggests that the cyclin F–EXO1 axis can indeed be dysregulated in GBM, validating the model used for the initial screen. It is possible that these and other mutations affecting IR-related pathways would be more evident in tumors that recur after radiotherapy, but this cannot be confirmed as repeat surgery is rare, limiting sample availability.

Together, the phenotypes induced by loss of cyclin F in the CCNF K/O reported above were recapitulated by expressing a nondegradable EXO1 R842A.

### EXO1 R842A mutant leads to hyper-resection and chromosome aberrations after IR

Our data support the idea that the regulation of EXO1 is mainly enacted by cyclin F during the cell cycle; however, the functional mechanism inducing defective DNA repair upon EXO1 expression in mitosis is unclear.

**Fig. 7.**
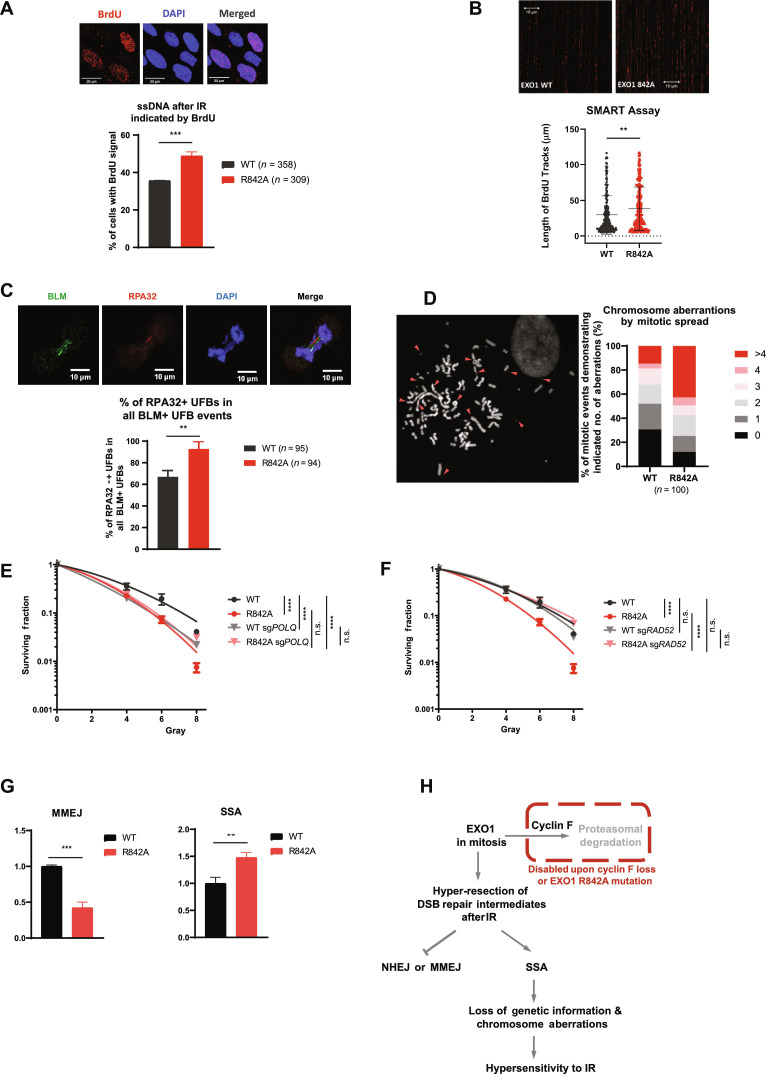
EXO1 R842A mutant leads to hyper-resection and toxic single-strand annealing after IR. (**A**) Representative images and quantification of native BrdU signal in IR-treated LN229 expressing HA-EXO1 WT or HA-EXO1R842A, as indicated. Error bars indicate SDs of three biological replicates. Two-tailed unpaired *t* test was performed as statistical analysis. ***P ≤ 0.001. (**B**) Representative images and quantification of the SMART assay in LN229 lines described in (A). >300 events were quantified. Error bars indicate SDs of three biological replicates. Mann-Whitney *U* test was used to derive significance. *P* = 0.0059, *U* = 17873.5, and *Z* = −2.7533. (**C**) Representative images and quantification of RPA32 positive anaphase ultrafine bridges (UFBs) in LN229 lines expressing HA-EXO1 WT or HA-EXO1R842A. Error bars indicate SDs of three biological replicates. Statistical analysis was performed using two-tailed unpaired *t* test. **P ≤ 0.01. (**D**) Quantification of chromosome aberrations in hTERT RPE-1 stably expressing HA-EXO1 or HA-EXO1 R842A mutant. A total of 100 metaphase events were analyzed for each condition. Percentages of mitotic events demonstrating 0, 1, 2, 3, 4, or >4 aberrations were calculated and plotted. (**E**) LN229 expressing HA-EXO1 WT or HA-EXO1 R842A transfected with Cas9 and sg*POLQ* or control sgRNA were treated with IR and subjected to colony formation assay. Statistical analysis derived with two-tailed unpaired *t* test. (**F**) LN229 cells expressing HA-EXO1 WT or HA-EXO1 R842A transfected with Cas9 and sg*RAD52* or control sgRNA were treated with IR and subjected to colony formation assay. Statistical analysis derived with two-tailed unpaired *t* test. (**G**) Quantification of MMEJ and SSA reporter assays as indicated. Repair efficiency was normalized against cells expressing control sgRNA. Statistical analysis was performed using two-tailed unpaired *t* test in three biological replicates. **P ≤ 0.01. (**H**) Schematic illustration of cyclin F–EXO1 axis controlling DSB repair pathway choice in mitosis.

EXO1 is crucial for DNA long-range end resection, a process that generates ssDNA upon DSB. Thus, we measured the formation of ssDNA as an indicator of EXO1 activity. As shown in fig. S7A, a substantial increase in the percentage of cells with ssDNA was observed in cells devoid of cyclin F (*CCNF K/O*) (fig. S7A). In cells lacking both cyclin F and EXO1, the formation of ssDNA was reduced to basal levels (fig. S7A), showing that the elevated ssDNA formation in these cells is due to EXO1-mediated resection. Similarly, more cells with ssDNA were detected in cells expressing EXO1 R842A mutant (Fig. 7A).

We also assessed DNA end resection directly at individual DNA molecules via SMART assay (single molecule analysis of resection tracks) (*38*). We found a substantial increase in the length of resection tracks in R842A mutant (Fig. 7B). Together, these results show that EXO1 stabilization can lead to an increase in both prevalence and length of DNA resection, a phenomenon known as DNA hyper-resection.

Our previous data show that the bulk of EXO1 degradation occurs in mitosis; thus, we moved on to assess the presence of ssDNA in mitosis as anaphase ultrafine bridges (UFBs) characterized by replication protein A2(RPA32) coating. UFBs are characterized by the localization of Bloom’s syndrome helicase (BLM) and PLK1-interacting checkpoint helicase to the ssDNA in unresolved recombination intermediates or at common fragile sites. UFBs coated with replication protein RPA32 represent stretches of unrepaired ssDNA, which eventually fracture at the end of mitosis (*39*, *40*). Examples of RPA32-positive and RPA32-negative UFBs are shown in the top of Fig. 7C. Quantification of these events in cells expressing EXO1 WT or EXO1 R842A showed a significantly higher incidence of RPA32-positive UFBs in EXO1 R842A mutant (Fig. 7C, bottom). Similarly, UFBs were increased in cells lacking cyclin F (*CCNF* K/O) (fig. S7B) and restored by concomitant *EXO1* depletion in the *CCNF* K/O background. The experiments above support the idea that both *CCNF* K/O and EXO1 R842A mutant lead to DNA hyper-resection in mitosis.

Hyper-resected DNA creates mechanically fragile sites in the genome, the fracture of which could eventually lead to chromosome breakage during segregation. Moreover, extensive resection may also expose single-stranded homologies among random genomic regions, which could facilitate unwanted homology recombination in between or within chromosomes and could lead to the formation of radial chromosome or chromosome fusion. This speculation is supported by the substantial higher incidence in chromosome aberrations observed in EXO1 R842A mutant–expressing hTERT RPE-1 cells after IR (Fig. 7D). In these cells, we could observe radial chromosomes, chromosome fusions, and chromosome breakages.

The presence of extensive DNA resection is a sign of uncontrolled EXO1 activity at DSBs. As extensive resection can prevent the efficient execution of multiple DSB repair pathways (*41*, *42*), we decided to identify DSB repair pathways that become defective upon aberrant EXO1 stabilization. To this end, we concomitantly genetically ablated HR, NHEJ, SSA, or MMEJ in cells expressing EXO1 WT or EXO1 R842A mutant and assessed IR sensitivity. After removing *XRCC4* (essential for NHEJ), *POLQ* (necessary for MMEJ), *RAD51* (important for HR), and *RAD52* (required by SSA) in cells expressing EXO1 WT or EXO1 R842A, respectively, deficiencies in NHEJ or HR were found to have an additive effect on the radiosensitization phenotype elicited by EXO1 R842A expression. In other words, removal of *XRCC4* or *RAD51* promotes IR sensitivity independently of EXO1 stabilization (fig. S7, C and D). On the contrary, the effect of removing *POLQ* was epistatic to EXO1 R842A expression, indicating that MMEJ is compromised in cells with high levels of EXO1 R842A (Fig. 7E). Genetic ablation of *RAD52* in EXO1 R842A cell line fully rescued the IR sensitivity promoted by EXO1 stabilization (Fig. 7F), showing that EXO1 stabilization leads to DNA damage and cell death through SSA. We measured the efficiency of the four major DSB repair pathways in cells expressing EXO1 WT or EXO1 R842A. In accordance with our hypothesis and the data obtained through survival assays, cells expressing EXO1 R842A had a reduced MMEJ and increased SSA compared to cells expressing EXO1 WT (Fig. 7G and fig. S7F). Overall, our findings show that the degradation of EXO1 in mitosis operated by SCF^cyclin F^ allows for the correct deployment of MMEJ.

## DISCUSSION

To identify determinants of IR sensitivity, we performed a focused and high-resolution CRISPR screen in radioresistant GBM cell lines. The screen identified both well-established and new factors modulating IR sensitivity. Among the latter, cyclin F depletion was validated to promote IR sensitivity. Subsequent investigation revealed that cyclin F ubiquitinates and mediates the proteasomal degradation of EXO1 in G_2_/M cell cycle phases. EXO1 accumulation upon cyclin F loss accounts for the IR hypersensitivity observed in *CCNF* K/O cells. EXO1 was previously identified as a substrate of cyclin F (*11*) upon DNA damage induction. Our study instead revealed a cell cycle–dependent EXO1 regulation by cyclin F. Moreover, we showed that cyclin F–EXO1 interaction requires CDK1/cyclin A–mediated phosphorylation at T824 as a priming signal, and the interaction is mediated by the cyclin domain on cyclin F and the RAIF motif at EXO1’s C terminus. Expression of the nondegradable R842A EXO1 mutant recapitulated the IR hypersensitivity phenotype observed in cells lacking cyclin F (*CCNF* K/O condition). Further investigation pointed out that the presence of EXO1 in G_2_/M promotes the formation of ssDNA and prevents the action of POLQ. Therefore, it is plausible that EXO1 in mitosis allows extensive resection at DSBs normally repaired by POLQ. The extensive resection in these regions prevents their repair by MMEJ as POLQ prefers limited end resection, while SSA requires long-range resection operated by EXO1 (*43*). Therefore, our findings on cell survival are in line with a model where cells attempt to repair the DSBs in mitosis using SSA but fail to execute SSA efficiently, due to the absence of a homologous template. Our finding on cyclin F–mediated EXO1 degradation in G_2_/M explains why MMEJ is mainly restricted in mitosis (*44*, *45*), establishing a mechanistic insights into the governance of the pathway choice between HR and MMEJ during cell cycle.

UBA3 inhibitor MLN4924 was previously reported to increase IR sensitivity in cells and in vivo (*46*). However, the individual CRL adaptors and the mechanisms responsible for such hypersensitivity remained unclear. Our screen identified multiple components of the Cullin family E3s, including but not limited to CUL1 and cyclin F, which opens several routes of investigation to explain the pleiotropic effects of MLN4924.

A key mediator of MMEJ is the DNA polymerase POLQ. Several POLQ inhibitors are being developed and are entering clinical trials as they demonstrate exciting anticancer potentials (*47*). Given the fact that *CCNF* depletion or EXO1 R842A mutant–mediated hyper-resection leads to defective MMEJ, exploiting the cyclin F–EXO1 pathway could be an alternative approach to POLQ inhibition to restrict MMEJ and, therefore, to sensitize cells to IR.

A crucial pathway that preserves the integrity of chromosomes in mitosis is operated by the cancerous inhibitor of protein phosphatase 2A (CIP2A) (*48*), which tethers chromosome ends to prevent breakage. In the future, it will be important to determine whether the cyclin F–EXO1 axis and the CIP2A axis co-operate or counteract each other to devise synthetic lethal approaches that exploit control of DNA repair in mitosis.

Modulation of the cyclin F–EXO1 axis could be used to induce formation of micronuclei, which are known to facilitate activation of the inflammatory signaling after IR and works synergistically with radiotherapy (*49*). Together these observations point out that cyclin F might be a promising target for increasing the efficacy of radiotherapy in glioblastoma and other tumor types.

We showed that the G_2_/M degradation of EXO1 is initiated by CDK1/cyclin A–mediated phosphorylation and is cell cycle dependent. We and others (*9*, *11*) also observed (Fig. 3D and fig. S3A) that SCF^cyclin F^ mediates EXO1 degradation upon IR, ultraviolet, and other genotoxic stimuli. Therefore, it is reasonable that additional regulatory phosphorylation events on EXO1 could exist and are required for DNA damage–induced EXO1 degradation.

Our study also focused on the analogy between degrons found in RRM2 and EXO1 and uncovered a recognition site specific to cyclin F and distinct from the classical Cy motifs (RxL or KxL). Our investigation reveals that cyclin F has a multivalent recognition site interacting directly with a threonine phosphorylated residue and an RxIF motif, which we name F-deg. The multivalency in substrate recognition allows a fail-safe recognition of substrates and resolves the conundrum of how cyclin F achieves substrate specificity instead of degrading all proteins containing Cy motifs. It is worth mentioning that our model does not exclude the possibility that cyclin F is still able to interact with the canonical Cy motif but defines how cyclin F specifically distinguishes substrate interactors from nonsubstrate interactors.

In addition to helping elucidate a pathway for the repair of DSBs after IR, our CRISPR screen highlighted many factors with unknown roles. Although the use of immortalized cell lines in this screen prevents true recapitulation of transcriptional features in human glioblastoma cells (*50*), these factors represent potential weaknesses of glioblastoma cells, exploitable in targeted therapies. Further expansion of the screen in glioma stem cells or in in vivo models shall validate the work presented here in a more clinically relevant context.

## METHODS

### Reagents, antibodies and cell lines

All reagents, Antibodies and cell lines used in this study are listed in Table 1.

**Table 1.** Reagents, antibodies, and cell lines used in the study. IgG, immunoglobulin G; GAPDH, glyceraldehyde-3-phosphate dehydrogenase; HRP, horseradish peroxidase.

Reagents	Source	Identifier
**Antibodies**		
Alexafluor 488 goat anti-rabbit IgG (H + L) cross-adsorbed secondary antibody	Invitrogen	A-11008
Alexafluor 568 goat anti-mouse IgG (H + L) cross-adsorbed secondary antibody	Invitrogen	A-11004
Anti-NEK9	Santa Cruz Biotechnologies	sc-100401
Anti-Flag agarose beads (affinity agarose gel)	Sigma-Aldrich	A2220-5ML
GFP-t + A3:C34rap agarose beads	Proteintech	gta-20
Mouse anti–α-tubulin	Santa Cruz Biotechnologies	sc-23948
Mouse anti-BrdU	BD Biosciences	347580
Mouse anti-Cas9	Cell Signaling Technology	14697T
Mouse anti-CUL1	Invitrogen	32-2400
Mouse anti-cyclin B	Invitrogen	MA5-13128
Mouse anti-E2F1	Santa Cruz Biotechnologies	A300-766A
Mouse anti-GAPDH	Invitrogen	MA5–15738
Mouse anti-GFP	Santa Cruz Biotechnologies	SC-9996
Mouse anti-HA	BioLegend	901501
Mouse anti-p53	Santa Cruz Biotechnologies	sc-126
Mouse anti-RPA32	Abcam	ab2175
Mouse anti-RPA32	Abcam	ab2175
Mouse anti-RRM2	Santa Cruz Biotechnologies	sc-398294
Mouse anti-ubiquitin	Novus Biologicals	NB300–130
Mouse anti-V5	eBioscience	14–6796-82
Rabbit anti-BLM	Abcam	ab2179
Rabbit anti–cyclin A	A gift from M. Pagano lab	N/A
Rabbit anti–cyclin F	Santa Cruz Biotechnologies	sc-952
Rabbit anti-EXO1	Bethyl	A302-640A
Rabbit anti-Flag	Sigma-Aldrich	F7425-.2MG
Rabbit anti-phospho CDK1 T14/Y15	Santa Cruz Biotechnologies	SC-12340
Rabbit anti-phospho H3 S10	Millipore	06-570
Rabbit anti-phospho RPA32 S33	Bethyl	a300-246A-M
Rabbit anti-phospho RPA32 S4/S8	Bethyl	A300-245A-T
Rabbit anti-SKP1	Cell Signaling Technology	2156S
Rabbit anti-ααH2Ax	Novus Biologicals	NB100-2280
Strptavidin-HRP	Thermo Fisher Scientific	N100
**Reagents, CRISPR library, and recombinant proteins**		
0.5 M EDTA pH 8.0 stock	AppliChem	A48920500
1 M magnesium chloride stock	Sigma-Aldrich	63069-100ML
1 M tris-HCl pH7.5 stock	VWR	E691-500 ml
1,10-Phenanthroline	Scientific Laboratory Supplies	CHE2730
4–12% bis-tris gradient precast gel	Thermo Fisher Scientific	NW04125BOX
4× Laemmli sample buffer	Invitrogen	NP0008
5 M NaCl stock	Gibco	24740011
Acetic acid glacial	Sigma-Aldrich	A6283-500ML
Acetonitrile	Sigma-Aldrich	271004-100ML
Ammonium bicarbonate	Fluka Analytical	09830-500G
Antifade mounting medium	VECTASHIELD	H-1000
ATP	Cell Signaling Technology	9804S
β-Glycerophosphate	Sigma-Aldrich	G5422
β-Mercaptoethanol	Thermo Fisher Scientific	31350010
Blasticidin S HCl	Cambridge Bioscience	14499-25 mg-CAY
BrdU	Cayman Chemical	CAY15580-500 mg
Colcemid	Gibco	15212012
Crystal violet	Alfa Aesar	B21932
CHX	Sigma-Aldrich	C7698-5G
DAPI	Sigma-Aldrich	MBD0015-1ML
Doxycycline	Sigma-Aldrich	D9891
DMEM	Sigma-Aldrich	D6429-500ML
Edit-R Human Lentiviral sgRNA Pooled Library for ubiquitin conjugation	Horizon Discovery	VSGH11110
Ethanol	Sigma-Aldrich	32221–2.5 L-M
FBS	Gibco	10500064
FiberPrep DNA Extraction Kit	Genomic Vision	EXT-001A
Formic acid	Merck	5.33002.0050
Glutathione sepharose beads	Cytiva	GE17-0756-01
Glycerol	Thermo Fisher Scientific	17904
HEPES	Sigma-Aldrich	H3375-500G
Hygromycin B	Thermo Fisher Scientific	10687010
KCl	Sigma-Aldrich	P9541-1KG
Kinase buffer	Cell Signaling Technology	9802S
λPP	New England Biolabs	P0753S
Lipofectamine 2000	Invitrogen	11668019
Methanol	Thermo Fisher Scientific	10164663
NEM	Sigma-Aldrich	E3876-5G
NaF	Fluka Analytical	S7920-100G
Nitrocellulose membrane	Amersham	10600006
Nocodazole	Selleckchem	S2775
Nonidet P-40 alternative	EMD Millipore	492016-100ML
Okadaic acid	Cayman Chemical	10011490-50 ug-CAY
Penicillin-streptomycin	Gibco	15140122
Phosphoric acid	Sigma-Aldrich	695017-100ML
PIPES	Sigma-Aldrich	P1851
PMSF	Santa Cruz Biotechnologies	sc-482875
PEI	Polysciences Inc.	23966
PR-619	ApexBio	A821
Protease inhibitor cocktail for mammalian cells	Sigma-Aldrich	P8340
Puromycin	Santa Cruz Biotechnologies	sc-108071
RIPA lysis buffer	Thermo Fisher Scientific	89901
Sodium deoxycholate	Sigma-Aldrich	D6750-10G
SDS	Sigma-Aldrich	74255-250G
Strep-Tactin Superflow sepharose beads	IBA Lifesciences	2-1208-002
Sucrose	Sigma-Aldrich	S0389-500G
Tetracycline-free FBS	PAN Biotech	P30-3602
Thymidine	Alfa Aesar	B21280
TEAB	Thermo Fisher Scientific	90114
TFA	Fisher chemical	T/3258/PB05
TCEP	Thermo Fisher Scientific	77720
Triton X-100	Sigma-Aldrich	T8787-250ML
Trypsin/Lys-C mix	Promega	V5071
Zeocin	Sigma-Aldrich	R25005
**Expression vectors**		
pCW57.1	Addgene	41393
pEGFP-C3	Clontech	6082-1
pOG44 Flp-Recombinase expression vector	Invitrogen	V600520
TurboID expression vector	Gift from Huber Lab	N/A
**Cell lines**		
LN229	ATCC	CRL-2611
HeLa	ATCC	CCL-2
HEK293T	ATCC	CRL-3216

### CRISPR screen and data analysis

Viral particles for the Edit-R Human Lentiviral sgRNA Pooled Library–UB conjugation (catalog no. VSGH11110) were purchased from Dharmacon. Viral particles were transduced in LN229 Cas9-stable line with a multiplicity of infection at 0.3 (less than 3% of all cells will be infected by more than one viral particle). We performed the transduction to ensure a fold of representation at 750 (meaning that, on average, the transduction of each guide was repeated in 750 different cells in the pooled population) and kept this fold of representation throughout the screen. Each sample was run in biological quadruplicates. After conducting the treatment, the genomic DNA was extracted using a Qiagen DNA extraction kit (catalog no./ID, 69504) according to manufacturer’s instructions. sgRNA sequences harbored were amplified by polymerase chain reaction (PCR) using Edit-R Pooled sgRNA Indexing PCR and Sequencing Primer Kit A (PRM10184) and Edit-R Pooled sgRNA Indexing PCR and Sequencing Primer Kit B (PRM10185). PCR products were sequenced on a Hiseq4000 at the Welcome Trust center for Human Genetics, Oxford. The sequencing data were converted in read count and statistical analyses were run using CRISPanalyzerR.

### Over-representation analysis

ORA was performed using the EnrichR program (*51*). In short, statistically significant genes conferring either IR sensitivity or resistance ([*z* ratio] > 1.96) were combined as one gene set and submitted for analysis on the EnrichR website. The dataset was ranked by κ score (*52*). Pathways with a κ score of ≥0.65 were considered pathways identified with high confidence from the screen in response to IR treatment. Pathways with a κ score of ≥0.5 but <0.65 were considered confident identifications, and those with a κ score of ≥0.35 but <0.5 were considered possible hits.

### Cell culture

LN229, HEK293T, H4, SW1088, DBTRG, A172, and HeLa cells were obtained from American Type Culture Collection (ATCC). All cell lines were cultured in Dulbecco’s modified Eagle’s medium (DMEM; Sigma-Aldrich, D6429-500ML) containing 10% fetal bovine serum (FBS; Life Technologies, 10500064) and the mixture of penicillin (100 U/ml) and streptomycin (100 μg/ml; Life Technologies, 15140122). HeLa cells knockout for *CCNF* and/or *EXO1* were generated using CRISPR (sgRNA sequences: table above). LN229 cells transduced with pCW57.1 lentiviral expression system were maintained in tetracycline-free media (DMEM plus 10% tetracycline-free FBS purchased from PAN Biotech, P30-3602). Expression of pCW57.1-EXO1 was induced by culturing the EXO1-stable cells in media containing doxycycline (Sigma-Aldrich, D9891, at a final concentration of 1 μg/ml) for 3 days before seeding for experiments. Doxycycline (1 μg/ml) was always present and was replenished every 3 days during experiments unless otherwise specified.

### Colony formation assay

Cells were counted and seeded at 400 cells per well in six-well plates and cultured for 6 hours before being challenged with the indicated IR doses using a closed source gamma irradiator. After IR treatment, cells were allowed to propagate for 7 days (for HeLa-derived cell lines) or 14 days (for LN229-derived cell lines). Colonies were fixed and visualized using crystal violet solution (50% methanol, 10% ethanol, and 0.3% crystal violet) for 10 min at room temperature with gentle agitation (10 to 20 rpm). After rinsing with water and airdrying, colonies were counted using GelCount mammalian-cell colony counter (Oxford OPTRONIX). All colony formation assays presented in this study have been repeated at least three times. And error bars represent SD of three biologically replicates. Statistical analysis was done using two-tailed unpaired *t* test.

### Generation of TurboID–cyclin F stable cell line

Flp-In T-REx HEK293 cells (Invitrogen, R78007) containing a single genomic FRT site and stably expressing the Tet repressor were cultured in DMEM supplemented with 10% FBS, zeocin (100 μg/ml), and blasticidin (15 μg/ml). The medium was exchanged with fresh medium containing no antibiotics before transfection. For cell line generation, Flp-In HEK293 cells were cotransfected with the pCDNA3–TurboID–cyclin F plasmid and the pOG44 Flp–recombinase expression vector (Invitrogen, V600520) for coexpression of the Flp-recombinase using Lipofectamine 2000 transfection reagent (Invitrogen, 11668019). Two days after the transfection, cells were selected in hygromycin-containing medium (100 μg/ml) for 2 to 3 weeks. To validate the TurboID–cyclin F expression, cells were cultured in media containing doxycycline (1.3 μg/ml) for 24 hours to induce TurboID–cyclin F expression before immunoblotting.

### MS sample preparation for TurboID–cyclin F pulldown

When TurboID–cyclin F Flp-In T-REx HEK293 cells grown in 15-cm dishes reached 80% confluency, doxycycline (1.3 μg/ml) was added for 24 hours to induce the expression of TurboID–cyclin F. Cells were further incubated with 50 μM biotin for 3 hours to label proteins that came into close proximity with TurboID–cyclin F in cells. Cells were harvested by scraping and washed three times with phosphate-buffered saline (PBS). For streptavidin pulldown of all biotin-labeled proteins (potential cyclin F interactors), cell pellets were thoroughly resuspended in 1 ml of RIPA buffer [50 mM tris-HCl (pH 8.0), 150 mM NaCl, 1% Triton X-100, 1 mM EDTA, and 0.1% SDS with protease inhibitor cocktail (Sigma-Aldrich, P8340)] and incubated on ice for 15 min. Insoluble material was removed by centrifugation. Cleared lysates were then incubated on a rotating wheel at 4°C with 50-μl pre-equilibrated Strep-Tactin Superflow Sepharose beads (IBA, 2-1208-002) for 1 hour. The suspension was then loaded on a Mini Bio-Spin Columns (Bio-Rad, 732-6207) to collect the beads. The beads were washed two times with 1 ml of RIPA buffer, three times with HNN buffer [50 mM HEPES (pH 7.5), 150 mM NaCl, and 50 mM NaF], and two times with 100 mM NH_4_HCO_3_ solution before being transferred to 2-ml Eppendorf tube in 400 μl of NH_4_HCO_3_ solution. For proteolysis, the sample was centrifuged at 200*g* for 1 min to remove supernatant. Beads were resuspended in 100 μl of 8 M Urea in 100 mM NH_4_HCO_3_ solution and incubated at 20°C for 20 min. Cysteine bonds were reduced with a final concentration of 5 mM tris(2-carboxyethyl) phosphine hydrochloride (TCEP) for 30 min at 37°C and alkylated in a final concentration of 10 mM iodoacetamide for 30 min at room temperature in the dark. Beads were then proteolyzed with trypsin/Lys-C Mix (Promega, V5071) at a 25:1 protein:protease ratio (w/w) for 4 hours at 37°C on an orbital shaker. Urea concentration was then reduced to 1 M via adding 100 mM NH_4_HCO_3_ solution to the sample. Samples were digested overnight at 37°C on an orbital shaker. Samples were desalted on C18 spin columns (Thermo Fisher Scientific, 89870) and washed according to the manual provided by the manufacturer and eluted with 0.1% trifluoroacetic acid (TFA) and 65% acetonitrile. Peptides were then dried in a SpeedVac vacuum concentrator and resuspend in 0.1% TFA and 2% acetonitrile in MS-grade water for MS analysis.

### LC-MS/MS sample preparation and data analysis

Please refer to the section above for MS sample preparation of the proximity labeling TurboID–cyclin F samples. For the identification of GFP-EXO1 WT versus T824A differential interactome, overexpressed GFP-EXO1 WT and T824A mutant were immunoprecipitated from HEK293T, respectively, and eluted using 2% SDS buffer. Eluents were then digested using S-Trap micro spin columns following the manufacturer’s protocol (Profiti, C02-micro-10). In brief, samples were reduced with 20 mM dithiothreitol and alkylated with 40 mM Iodoacetamide (30 min each at room temperature in the dark). Samples were then acidified with phosphoric acid (1.2% final concentration) and mixed with binding buffer [100 mM triethylammonium bicarbonate (TEAB) in 90% methanol] to a 1:7 ratio (sample:binding buffer). Samples were then transferred onto the S-trap spin column. Proteins in the samples were adsorbed onto columns via centrifugation before being washed with binding buffer for five times. Twenty microliters of 50 mM TEAB containing 1 μg of trypsin was then added to the column and incubated overnight at 37°C. Peptides were eluted with 50 mM TEAB and 2% formic acid in 50% acetonitrile solution and dried using a SpeedVac vacuum concentrator. Before MS, samples were resuspended in 0.1% TFA and 2% acetonitrile in MS-grade water.

Both TurboID–cyclin F and GFP-EXO1 MS, samples were analyzed by reverse-phase chromatography using an UltiMate 3000 UHPLC connected to an Orbitrap Fusion Lumos (Thermo Fisher Scientific). Peptides were trapped onto a PepMap C18 trap column (100 μm × 2 cm, 5 μm particle size; Thermo Fisher Scientific) and separated on a 50-cm Easy-Spray column (ES903, Thermo Fisher Scientific) using a 60-min linear gradient from 2 to 35% buffer B [buffer A: 5% dimethyl sulfoxide (DMSO) and 0.1% formic acid; buffer B: 5% DMSO and 0.1% formic acid in acetonitrile] at 250 nl/min flow rate. Eluted peptides were then analyzed in the Orbitrap Fusion Lumos in data-dependent mode with the advance peak detection switched on. Full scans were acquired in the Orbitrap at 120k resolution over a mass/charge ratio (*m*/*z*) range of 400 to 1500, Automatic Gain Control target of 4 × 10^−5^, and S-lens radio frequency of 30. MS2 scans were obtained in the ion trap (rapid scan mode) with a quad isolation window of 1.6, 40% AGC target, and a maximum injection time of 35 ms, with High Collision Dissociation activation and 28% collision energy.

The raw MS data were analyzed using MaxQuant (v1.6.14). Briefly, files were searched against the UniProt-Swissprot human database using the built-in Andromeda data-search engine. Trypsin was selected as enzyme (up to two missed cleavages), carboamidomethylation (C) as fixed modification and deamidated (NQ) and oxidation (M) as variable modifications. Protein false discovery rate was set up at 1%. Data were quantified using the label-free quantitation and the intensity-based absolute quantification (iBAQ) parameter was enabled. Match between runs was not selected. Maxquant protein group output was further analyzed using Perseus (1.6.2.2). For TurboID–cyclin F dataset (*n* = 3 per condition), intensities were log 2 transformed and normalized by median subtraction before a 20% total valid number filter was applied. Missing values were imputed (following a down-shifted normal distribution). A two-sample Student’s *t* test was applied in combination with a permutation–FDR correction (5%). For EXO1 WT versus T824A interactome dataset (*n* = 1 per condition), iBAQ values were log 2 transformed and missing values were replaced by a constant equal to 5.

The MS raw data included here has been deposited to the Proteome eXchange Consortium via the PRIDE partner repository with the dataset identifier PXD051841 (*53*).

### CRISPR knockout

Stable knockout was generated by cotransfecting Cas9 protein and three sgRNAs targeting the same gene, designed by Synthego, using Lipofectamine CRISPR Max (Life Technologies, CMAX00001) according to the protocol here: https://www.synthego.com/products/crispr-kits/synthetic-sgrna. Four days after transfection, cells were trypsinized and seeded as single cells in 96-well plates to isolate single clones. Cells in wells with proliferative clones were then trypsinized and expanded in bigger vessel until there were enough cells for immunoblotting. Clones that showed clear knockout were further validated by genomic DNA extraction and PCR amplification of the exon targeted by the sgRNAs. The PCR product was ligated into pCR4 vector using a TOPO TA cloning kit (Invitrogen, 450030). After transforming into DH5α competent cells, 10 colonies were picked and sent for Sanger sequencing. In LN229, cells were cotransfected with Cas9 protein and Synthego sgRNAs and directly subjected to treatment and western blotting (WB) 4 days after transfection. Although single clone picking was not performed, knockout efficiency in the mixed population was proven to be adequate (Fig. 3D).

### Transfection, IP, and WB

A total of 1.5 million HEK293T cells were seeded into each of the 10-cm petri dish 24 hours before plasmid transfection. For each 10-cm dish, the polyethylenimine linear (PEI) transfection was performed by vigorously vortexing 5 μg of plasmid DNA with 15 μl of PEI (2.5 mg/ml) in 400 μl of plain DMEM (without FBS or antibiotics) for 15 s to mix, incubating the mixture for 15 min at room temperature, then adding it to cells cultured in 10-ml complete media in a dropwise fashion. PEI was purchased from Polysciences Inc. (23966). PEI stock (2.5 mg/ml) was made in 20 mM HEPES, 150 mM NaCl (pH 7.4), and filtered. Twenty-four hours after transfection, cells were washed twice with PBS and harvested. The cell pellets were stored at −80°C or lysed directly for experiments.

Cell pellets harvested for IP or WB not aiming to detect DNA damage markers were lysed in lysis buffer containing 50 mM tris-HCl (pH 7.5), 150 mM NaCl, 1 mM EDTA, 5 mM MgCl_2_, and 0.1% Nonidet P-40, supplemented with protease inhibitor cocktail (Sigma-Aldrich, P8340), 200 μM phenylmethylsulfonyl fluoride (PMSF; Santa Cruz Biotechnologies, sc-482875), and two phosphatase inhibitors, 20 mM β-glycerophosphate (Sigma-Aldrich, G5422) and 1 μM Okadaic acid (Cayman Chemical, 10011490-50 ug-CAY). After lysing on ice for 10 min, the insoluble fraction (mostly DNA and DNA bound proteins) was removed via centrifugation at 20,000*g* at 4°C for 15 min, and the supernatant was carefully transferred to new Eppendorf tubes without disturbing the insoluble fraction. Protein concentration of the supernatant was measured using the modified Lowry assay (DC Protein Assay Kit, Bio-Rad, 5000111). The same amount of total protein was used for each IP (0.5 to 1 mg per pulldown in general) or direct WB (10 to 20 μg per lane in general). Final samples were mixed with 4X Laemmli sample buffer (Invitrogen, NP0008) and boiled for ten minutes before being applied in SDS-PAGE. For IP, 10-μl Flag M2 beads (Sigma-Aldrich, A2220-5ML) or 15-μl HA beads (Sigma-Aldrich, E6779-1ML) were washed three times with lysis buffer and added to the cell lysates to incubate for 3 hours on a roller at 4°C. After incubation, the beads were collected by centrifugation and washed with inhibitor-containing lysis buffer five times before being mixed with 20 to 40 μl 1× Laemmli sample buffer (diluted from Invitrogen, NP0008) supplemented with β-mercaptoethanol, and boiled for 10 min at 95°C. The boiled supernatant can then be applied in WB analysis.

For experiments detecting DNA damage markers (which could be tightly chromatin bound), cell pellets were harvested, homogenized, and boiled directly in 2% SDS buffer [350 mM bis-tris (pH 6.8), 20% glycerol, and 2% SDS], then sonicated. Protein concentration was assessed using a BCA protein kit (Thermo Fisher Scientific, 23227). Cell lysate was prepared for WB as indicated above and resolved in 4 to 12% gradient bis-tris gels, transferred onto nitrocellulose membrane (Amersham, 10600006) and immunoblotted. WB results were visualized via x-ray film or iBright FL1500 Imaging System (Invitrogen, A44241).

### Cell cycle synchronization via double thymidine release

A total of 500,000 cells were seeded into each 10-cm dishes. For HeLa and *CCNF* K/O cell lines, cells were seeded directly into the first thymidine block (2 mM final concentration) and cultured for 16 hours. After being washed three times with PBS and once with complete media, cells were released into fresh complete media for 8 hours. A second thymidine block was then performed with the same conditions as the first. Sixteen hours later, 0-hour time points were collected before the rest of the cells were washed and released into fresh complete media. Cells were collected at different time points as indicated. At the 7-hour time point, 200 nM nocodazole was added to the uncollected cells to prevent cells from entering the next cell cycle. For cell cycle synchronization of LN229, all conditions were kept the same except the duration of the two rounds of thymidine block, which changed from 16 to 24 hours.

### CHX chase

A total of 250,000 cells were seeded into each well of a six-well plate. Sixteen hours afterward, cells were cultured with CHX (50 μg/ml) for various durations as indicated in the figures, to block ribosomal protein synthesis for assessment of protein stability. Cells were collected via scraping and washed with PBS twice before being subjected immediately to WB for protein half-life estimation.

### In vivo ubiquitination assay

Two million HEK293T cells were seeded into each of the 10-cm dishes 24 hours before being transfected with plasmids as indicated in each experiment (typically, for each 10-cm dish, 1 μg of substrate overexpression plasmid, 2 μg of E3 overexpression plasmid, or 3 μg of ubiquitin overexpression plasmid was cotransfected). Cells were cultured for another 24 hours before being harvested. Four hours before the harvest, MG132 was added to a final concentration of 10 μM to block proteasomal degradation so that ubiquitination events were enriched. Cells were collected by scrapping and washed with PBS once, then thoroughly lysed and boiled in 300 μl of ubiquitin lysis buffer [2% SDS, 150 mM NaCl, and 10 mM tris-HCl (pH 7.4)]. After cooling to room temperature, cell lysates were subjected to sonication until they lost their viscosity. Lysates were then boiled again and centrifuged at 17,000 g for 10 min. Twenty μl of supernatant was preserved as input for each sample. The rest of the supernatant was diluted 20 times with dilution buffer [10 mM tris-HCl (pH 7.4), 150 mM NaCl, 2 mM EDTA, and 1% Triton X-100] and processed by IP of the substrate. After 16 hours of incubation on a roller at 4°C, beads were collected via centrifugation at 2000 rpm for 1 min and washed five times with 1 ml wash buffer [10 mM tris-HCl (pH 7.4), 1 M NaCl, 1 mM EDTA, and 1% Nonidet P-40] before being mixed with 50 μl of 1X Laemmli sample buffer, boiled, and subjected to WB.

### Ubiquitin binding entities pulldown assay

Before harvesting the cells, GST-UBA[ubiquitin-associated domain (UBA domain) of the UBQLN1 protein] was first conjugated to glutathione sepharose beads (Cytiva, GE17-0756-01) in ubiquitin binding entities (UBE) lysis buffer [19 mM NaH_2_PO_4_, 81 mM Na_2_HPO_4_ (pH 7.4), 1% Nonidet P-40, 2 mM EDTA, supplemented with protease inhibitor cocktail, PMSF, and phosphatase inhibitors as mentioned in the IP section], 50 mM *N*-ethylmaleimide (NEM; Sigma-Aldrich, E3876-5G), 5 mM 1,10-phenanthroline (Scientific Laboratory Supplies, CHE2730), and 50 μM PR-619 (ApexBio, A821) for at least 4 hours at 4°C on a roller. For each pulldown, 100 μg of recombinant GST-UBA was conjugated to 20 μl of washed glutathione beads. Cells were treated with 10 μM MG132 for 4 hours before being harvested by scrapping and centrifugation. Cell pellets were washed twice with PBS, then directly lysed in freshly prepared UBE lysis buffer. After incubation on ice for 10 min, lysates were subjected to centrifugation at 14,000 rpm at 4°C for 15 min. After the protein concentration was measured via Lowry assay using DC Protein assay kit (Bio-Rad, 5000111), the same amount of total protein was used for each pulldown (2 to 3 mg per pulldown). GST-UBA–conjugated beads were added to the lysate and incubated on a roller at 4°C for overnight, then collected, washed using UBE lysis buffer, mixed with 1× Laemmli sample buffer, and boiled in the same way as described in the IP section. The supernatant was then used for WB. Besides the protein of interest, total ubiquitin was probed as a loading control for UBE pulldown experiments.

### In vitro dephosphorylation

In vitro dephosphorylation was performed using λPP kit (New England Biolabs, P0753S). To remove phosphorylation on all proteins IP purified via agarose beads, after IP, beads were washed three times with lysis buffer containing only protease inhibitors but not phosphatase inhibitors and one time with 1× λPP buffer provided in the kit. After removing the wash buffer, beads were resuspended in 100 μl of 1× λPP buffer containing 1 mM MnCl_2_ and 400 U of λPP and incubated at 30°C, 800 rpm, for 1 hour on an orbital shaker. After the incubation, the beads were washed three times with 1× λPP buffer before being used for WB or for in vitro phosphorylation assay.

### In vitro phosphorylation assay

For in vitro kinase assays, enhanced green fluorescent protein (EGFP)–tagged EXO1 was overexpressed in HEK293T cells and purified by IP using GFP-Trap beads (Proteintech, gta-20). Before being used as the substrate, EGFP-EXO1–bound beads were first dephosphorylated by λPP (New England BioLabs, P0753S) to remove all existing phosphorylation. After washing off the λPP extensively with kinase buffer (Cell Signaling Technologies, 9802S), beads were incubated with 100 ng of CDK1/2–cyclin A/B/E (as indicated in Fig. 4, G and H) in kinase buffer supplemented with 200 μM adenosine 5′-triphosphate ATP for 30 min at 30°C. The reactions were stopped by mixing the reaction mixture with 4× Laemmli sample buffer and boiling at 95°C for 10 minutes. EXO1 T824 phosphorylation was visualized via SDS-PAGE and WB using the custom-made site-specific phosphorylation antibody described below.

### Generation of site-specific phosphorylation antibody targeting pT824 EXO1

Antibodies were generated by YenZym Antibodies LLC: The following pThr824–EXO1 peptide was synthesized and conjugated to a carrier protein through the N-terminal cysteine residue (Ahx is added as a linker). As a negative control for validation experiments, peptide with the same sequence, but no phosphorylation was also synthesized. Two rabbits were used for immunization. Twenty-one days after immunization, their sera was harvested and subjected to affinity absorption and ELISA validation. pThr824 EXO1 peptide C-Ahx-RDNIQLpTPEAEED-amide (amino acid residues from 818 to 830) and nonphosphorylated Thr^824^ EXO1 peptide C-Ahx-RDNIQLTPEAEED-amide were designed and synthesized by YenZym Antibodies LLC.

### In situ detection of ssDNA

The protocol used in this study was a modification of the one described in (*54*). In short, to visualize and quantify ssDNA generated from DNA resection, EXO1 expression in LN229 cells with pCW57.1-EXO1 WT or R842A was induced for 3 days in complete media containing doxycycline (1 μg/ml) before 300,000 cells per well were seeded in a six-well plate pre-filled with sterile glass coverslips (also with doxycycline). After the cells attached to the coverslips, 10 μM BrdU (Cayman Chemical, CAY15580-500 mg) was added, and the cells were incubated for 24 hours. BrdU-containing media was then replaced with fresh complete media containing no BrdU but doxycycline. Cells were then treated with 10-Gy IR and allowed to recover for 3 hours before being subjected to in situ fractionation on ice as follows: 10 min in pre-extraction buffer 1 [10 mM PIPES (pH 7.0), 300 mM sucrose, 100 mM NaCl, 3 mM MgCl2, and 0.5% Triton-X100], then 10 min in pre-extraction buffer 2 [10 mM tris-HCl (pH 7.5), 10 mM NaCl, 3 mM MgCl2, 1% Nonidet P-40, and 0.5% sodium deoxycholate]. Cells on coverslips were washed three times with PBS before being fixed with 4% paraformaldehyde for 15 min at room temperature. The cells were then washed with PBS and blocked/permeabilized in 3% bovine serum albumin (BSA) and 0.5% Triton X-100 dissolved in PBS for 30 min. Coverslips were incubated overnight at 4°C with mouse anti-BrdU antibody (1:500 dilution; BD Biosciences, 347580) under nondenaturing conditions followed by anti-mouse secondary antibody (1:1000 dilution; Invitrogen, A-11004) for 1 hour. As the anti-BrdU antibody detects BrdU incorporated into ssDNA but not dsDNA, the BrdU signal detected via this protocol is a good reflection of ssDNA generated by DNA resection. Following the antibody incubation, the cells were washed five times in 0.2% Triton X-100 in PBS before being mounted onto slides using VECTASHIELD antifade mounting medium (H-1000) supplemented with 4′,6-diamidino-2-phenylindole (DAPI; 5 μg/ml) and visualized using a Zeiss LSM 780 Confocal Microscope at 400× magnification.

### SMART assay

On average, ~1,000,000 cells per 10-cm dish were seeded for each sample in the experiment. After the cells attached, 10 μM BrdU (Cayman Chemical, CAY15580-500 mg) was added to the cell culture and incubated for 24 hours. BrdU-containing media was then switched to fresh complete media containing doxycycline but not BrdU. Cells were treated with 10-Gy IR and allowed to recover for 3 hours before being harvested by trypsinization, counted, and embedded in low-melting-point agarose gel plugs. About 250,000 cells were used for each plug. DNA extraction in the plug, agarose digestion of the plug, and combing were performed following the instructions in the kit manual of a FiberPrep DNA extraction kit (Genomic Vision, EXT-001A). BrdU was detected by 1-hour incubation of mouse anti-BrdU antibody (1:10 dilution, BD Biosciences, 347580) at 37°C followed by 1-hour incubation of the Cy3.5-conjugated anti-mouse secondary antibody (1:10 dilution; Abcam, AB6946) at 37°C under nondenaturing conditions in a wet chamber. Coverslips were scanned using the automated FibreVisionS scanner. Results were quantified using FiberStudio.

### Anaphase UFB detection and micronuclei counting

After treating cells with doxycycline for 3 days, cells were seeded at 200,000 cells per well in six-well plates filled with sterile coverslips. Twenty-four hours after seeding, cells were treated with 10-Gy IR and allowed to recover for 12 hours. Cells on coverslips were fixed and permeabilized with UFB buffer (4% paraformaldehyde in 20 mM PIPES at pH 6.8, 1 mM MgCl2, 10 mM EGTA, and 0.2% Triton X-100) for 10 min at room temperature. Samples were then washed with PBS containing 0.2% Triton X-100 for 5 min and blocked by 3% BSA for 30 min. Primary antibodies (Rabbit anti-BLM from Abcam ab2179 and mouse anti-RPA32 from Abcam ab2175) diluted in 3% BSA were then applied on top of each coverslip in a wet chamber and incubated at 4°C overnight. Next day, the unbound primary antibodies were washed away using 0.2% Triton X-100 in PBS for three times before being incubated with secondary antibodies (A-11004 and A-11008 from Invitrogen) diluted with 3% BSA for 2 hours at room temperature. Coverslip were washed with PBS for three times before being mounted onto glass slide using VECTASHIELD antifade mounting medium (H-1000) supplemented with DAPI (5 μg/ml). UFBs and micronuclei were visualized and counted using a Zeiss LSM 780 confocal microscope at 630× and 200× magnification, respectively.

### Mitotic spreading

EXO1 expression in hTERT RPE-1 cells containing pCW57.1-EXO1 WT or R842A constructs was induced for 3 days in complete media containing doxycycline (1 μg/ml) before 500,000 cells per 10-cm dish were seeded for the experiment (also with doxycycline). Twenty-four hours after reseeding, cells were treated with 10-Gy IR and allowed to recover for 3 days. Three hours before harvesting, cells were treated with colcemid (0.02 μg/ml; Gibco, 15212012) to enrich metaphase events. At the end of the colcemid treatment, media was collected to preserve the floating mitotic cells, and the adherent cells were also harvested via trypsinization. The floating and adherent cells were combined and thoroughly resuspended in 10 ml of hypotonic solution (75 mM KCl in water) and incubated at 37°C for 30 min. All cells were then collected via centrifugation at 200*g* for 10 min. The supernatant was removed until roughly 300 μl was left. Cells were then thoroughly resuspended in the remaining supernatant by flicking (not pipetting). Five ml of freshly made fixative (methanol: glacial acetic acid at 3:1 ratio) was then added to the supernatant in a dropwise fashion (flicked to mix after every drop). The fixation was performed on ice for 30 min. Fixed cells were once again centrifuged and resuspended in 300-μl residual supernatant, mixed with 5-ml fixative, and centrifuged again. The same process was repeated at least five times until hypotonic solution was replaced with fixative. After the last centrifugation, the cell pellet was thoroughly resuspended in approximately 200-μl residual fixative and kept on ice. Twenty μl of the cell suspension was dropped on a clean glass slide from a 10- to 15-cm vertical distance for metaphase spreading. The slide was then air-dried for 2 hours at room temperature before being mounted by VECTASHIELD antifade mounting medium (H-1000) supplemented with DAPI (5 μg/ml) and visualized using a Zeiss LSM 780 confocal microscope at 630× magnification.

### Cyclin F–EXO1 model

A computational protein-protein docking method was used to predict the atomic interactions between cyclin F and EXO1. “COSMIC^2^” served as the platform to determine cryo–electron microscopy structure and the tool used for prediction was AlphaFold2. The inputs included accession number NP_001752.2 for cyclin F and the sequence “LEPEAEEDIFNKPECGRVQRAIFQ” for EXO1. The parameters were set to consider “full database,” “multimer,” and “no relaxed” models. Five multimer models were generated and confidence scores of iptm + ipm were compared. ChimeraX was used to visualize the chosen model. The model had an iptm + ptm value of 0.7984765224933627.

### DSBs repair reporter assay

pCW57.1-EXO1 WT or R842A was introduced freshly before every experiment to the already puromycin-resistant HR, NHEJ, MMEJ, and SSA reporter assay cells using lentiviral transduction. To enhance transduction efficiency, virus packaged in HEK293T cells were concentrated using a sterilized 30-kDa centrifugal filter unit (EMD Millipore, UFC903024) before being applied to the target cells. Twenty-four hours after transduction, cells in each well of a six-well plate were transfected with 5 μg of I-SceI endonuclease expression plasmid mixed with 12 μl of Lipofectamine Stem reagent (STEM00015, Invitrogen) to initiate site-specific DSBs. Six hours after transfection, Opti-MEM media was switched back to puromycin-containing complete media, and EXO1 expression in these cells were induced by doxycycline (1 μg/ml) for 3 days before being harvested via trypsinization and subjected to flow cytometric analysis using the Attune NxT Flow Cytometer (Invitrogen).
