# Supplementary material for: Cyclin F–EXO1 axis controls cell cycle–dependent execution of double-strand break repair
Source: Sci Adv. 2024 Aug 9;10(32):eado0636. doi: 10.1126/sciadv.ado0636 (PMC11313846; doi:10.1126/sciadv.ado0636)
Supplement: Supplementary file 1 — Figs. S1 to S7 Legends for tables S1 to S4 [file sciadv.ado0636_sm.pdf]

Science Advances
AAAS

Supplementary Materials for

Cyclin F–EXO1 axis controls cell cycle–dependent execution of double-strand break repair

Hongbin Yang *et al.*

Corresponding author: Vincenzo D'Angiolella, vincenzo.dangiolella@oncology.ox.ac.uk,vdangio@ed.ac.uk

Sci. Adv. **10**, eado0636 (2024)
DOI: 10.1126/sciadv.ado0636

The PDF file includes:

Figs. S1 to S7
Legends for tables S1 to S4

Other Supplementary Material for this manuscript includes the following:

Tables S1 to S4

Figure S1

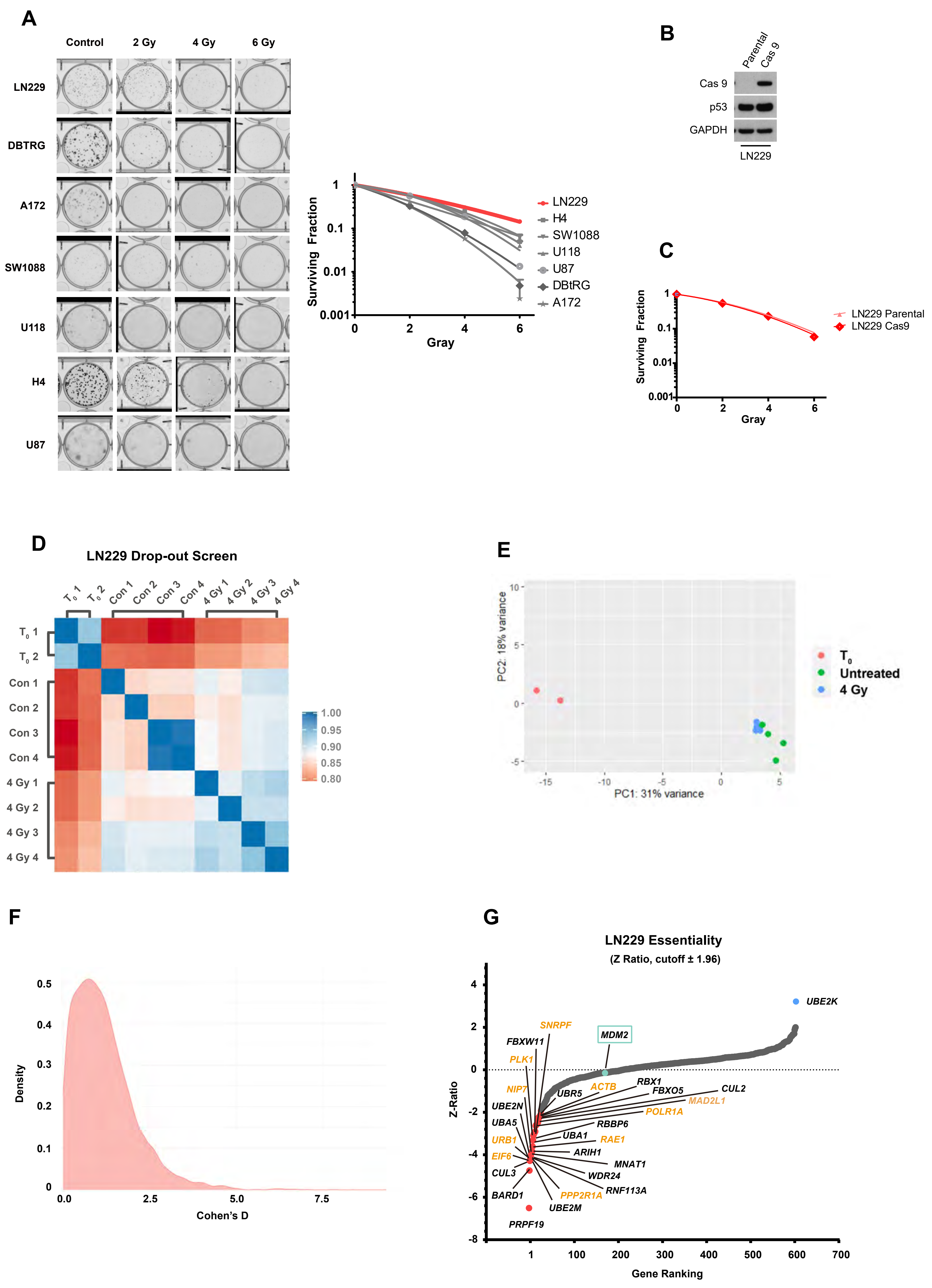

A
Control
2 Gy
4 Gy
6 Gy
LN229
DBTRG
A172
SW1088
U118
H4
U87
Surviving Fraction
1
0.1
0.01
0.001
0
2
4
6
Gray
LN229
H4
SW1088
U118
U87
DBtRG
A172
B
Parental
Cas 9
Cas 9
p53
GAPDH
LN229
C
Surviving Fraction
1
0.1
0.01
0.001
0
2
4
6
Gray
LN229 Parental
LN229 Cas9
D
LN229 Drop-out Screen
T0 1
T0 2
Con 1
Con 2
Con 3
Con 4
4 Gy 1
4 Gy 2
4 Gy 3
4 Gy 4
1.00
0.95
0.90
0.85
0.80
E
PC2: 18% variance
10
5
0
-5
-15
-10
-5
0
5
PC1: 31% variance
T0
Untreated
4 Gy
F
Density
0.5
0.4
0.3
0.2
0.1
0
0.0
2.5
5.0
7.5
Cohen's D
G
LN229 Essentiality
(Z Ratio, cutoff ± 1.96)
Z-Ratio
4
2
0
-2
-4
-6
-8
1
100
200
300
400
500
600
700
Gene Ranking
UBE2K
MDM2
SNRPF
FBXW11
PLK1
NIP7
UBR5
ACTB
RBX1
FBXO5
CUL2
MAD2L1
POLR1A
UBE2N
UBA5
RBBP6
UBA1
URB1
RAE1
ARIH1
EIF6
MNAT1
CUL3
WDR24
RNF113A
PPP2R1A
BARD1
UBE2M
PRPF19

Figure S1. Optimization and quality control of the CRISPR screen

A. Seven different GBM cell lines (LN229, DBTRG, A172, SW1088, U118, H4, U87) were seeded for colony formation assay and challenged with the indicated doses of IR. 14 days after IR, cells were stained with crystal violet and counted. Error bars represent standard deviations of three biological replicates.
B. Immunoblotting of a single cell clone derived from LN229 cells stably expressing Cas9 after lentiviral transduction;
C. Cells in B. were seeded for colony formation assay and challenged with the indicated doses of IR. 14 days after IR, cells were stained with crystal violet and counted.Error bars represent standard deviations of three biological replicates.
D. Rank correlation of normalized sgRNA read counts between biological replicates and treatment conditions of the CRISPR screen.
E. Principal component analysis (PCA) of samples for the CRISPR screen. Sample T0 is in orange, quadruplicated samples for untreated condition are in green, and 4 Gray IR treated samples are in blue.
F. Density plot depicting the effect size Cohen's d values between treated and untreated samples for each CRISPR guide.
G. Ranked genes using *z-ratio* after comparing untreated LN229 to T0 sample. Genes with negative *z-ratio* are considered essential genes (in red). Gene with positive *z-ratio* are in blue. A ±1.96 Z-ratio value was used as cut-off for significance. Gene names in orange indicates core essential positive control genes provided in the sgRNA library.

Figure S2

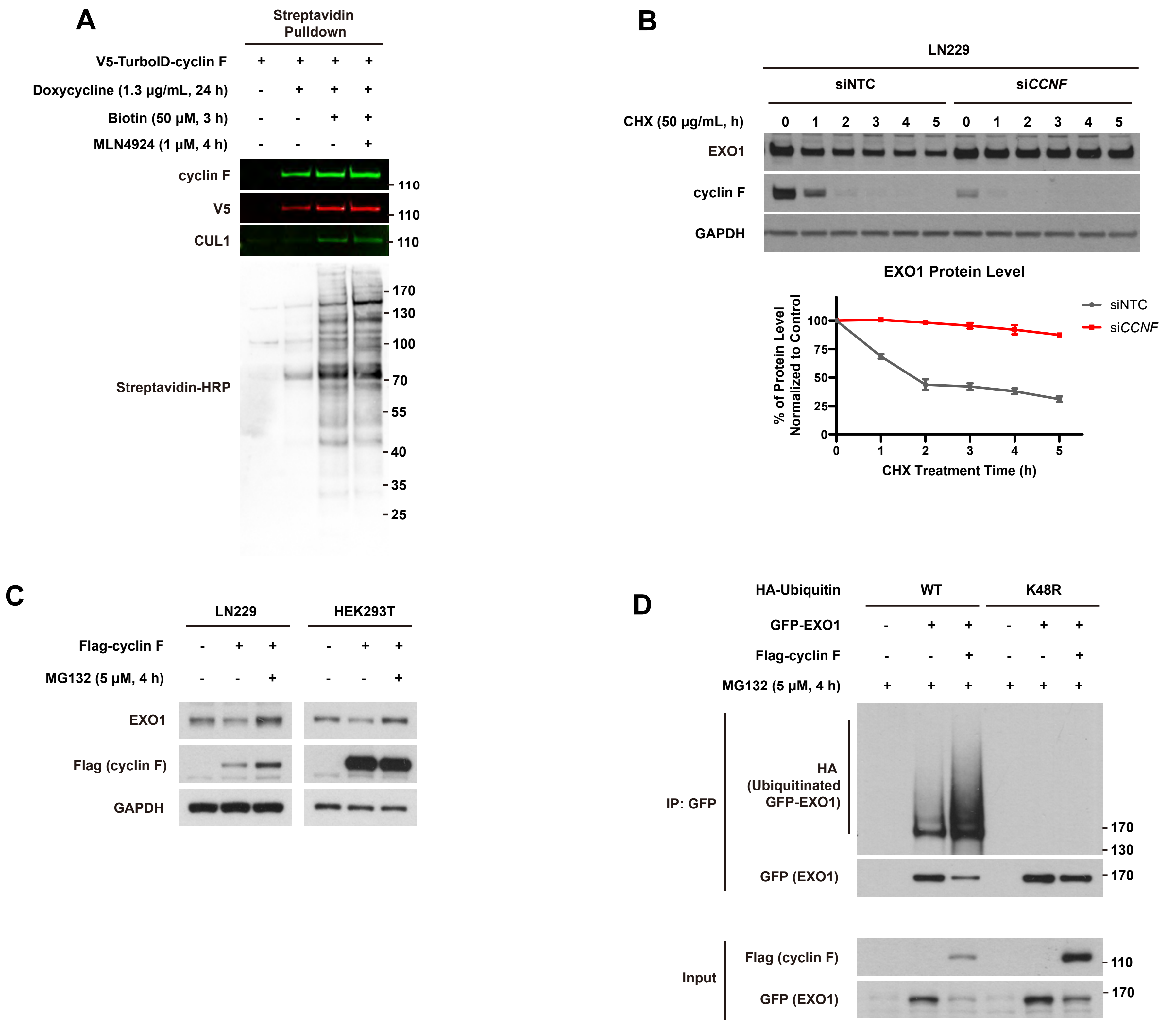
A
Streptavidin Pulldown
V5-TurboID-cyclin F
Doxycycline (1.3 µg/mL, 24 h)
Biotin (50 µM, 3 h)
MLN4924 (1 µM, 4 h)
cyclin F
V5
CUL1
Streptavidin-HRP
110
170
130
100
70
55
40
35
25
B
LN229
siNTC
siCCNF
CHX (50 µg/mL, h)
EXO1
cyclin F
GAPDH
EXO1 Protein Level
% of Protein Level Normalized to Control
CHX Treatment Time (h)
siNTC
siCCNF
C
LN229
HEK293T
Flag-cyclin F
MG132 (5 µM, 4 h)
EXO1
Flag (cyclin F)
GAPDH
D
HA-Ubiquitin
WT
K48R
GFP-EXO1
Flag-cyclin F
MG132 (5 µM, 4 h)
IP: GFP
HA (Ubiquitinated GFP-EXO1)
GFP (EXO1)
Input
Flag (cyclin F)
GFP (EXO1)
170
130
110

Figure S2. Cyclin F ubiquitinates and degrades EXO1

A. Immunoblotting after isolation of biotinylated proteins from HEK293T cells expressing TurboID-cyclin F after induction with doxycycline for 24 h. Cells were doxycycline induced and treated with biotin and/or MLN4924 as indicated. Biotinylated proteins were detected by HRP-conjugated streptavidin- *bottom panel*
B. Immunoblotting after transfection of LN229 cells with non-targeting control (NTC) siRNA or siRNA targeting *CCNF* treated with Cycloheximide (CHX) for the indicated time - *upper panel*. Relative quantification of EXO1 protein levels in cells after normalization against EXO1 levels at T0 for each cell line - *bottom panel*.
C. Immunoblotting of LN229 and HEK293T after transient transfection of Flag-cyclin F and treatment with MG132 as indicated.
D. Immunoblotting after expression of GFP-EXO1, Flag-cyclin F, HA-ubiquitin wild-type or K48R mutant in HEK293T. GFP-EXO1 is isolated *via* GFP beads pulldown after denaturation. Input samples before immunoprecipitation are indicated.

Figure S3

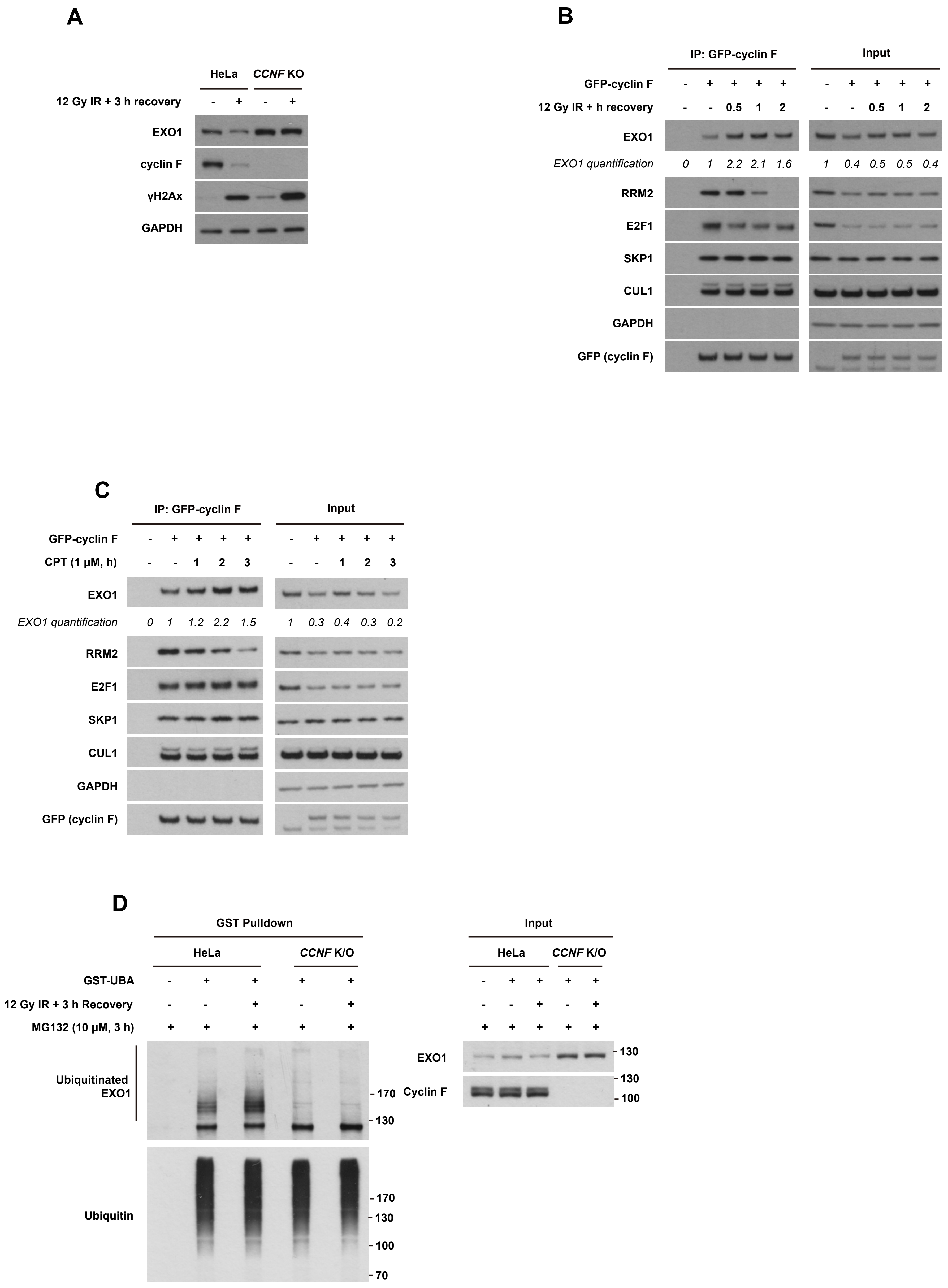
A
HeLa
CCNF KO
12 Gy IR + 3 h recovery
EXO1
cyclin F
γH2Ax
GAPDH
B
IP: GFP-cyclin F
Input
GFP-cyclin F
12 Gy IR + h recovery
0.5 1 2
EXO1
EXO1 quantification
0 1 2.2 2.1 1.6
1 0.4 0.5 0.5 0.4
RRM2
E2F1
SKP1
CUL1
GAPDH
GFP (cyclin F)
C
IP: GFP-cyclin F
Input
GFP-cyclin F
CPT (1 μM, h)
1 2 3
EXO1
EXO1 quantification
0 1 1.2 2.2 1.5
1 0.3 0.4 0.3 0.2
RRM2
E2F1
SKP1
CUL1
GAPDH
GFP (cyclin F)
D
GST Pulldown
Input
HeLa
CCNF K/O
GST-UBA
12 Gy IR + 3 h Recovery
MG132 (10 μM, 3 h)
Ubiquitinated EXO1
Ubiquitin
EXO1
Cyclin F
170
130
100
70

Figure S3. Cyclin F-mediated EXO1 ubiquitination is induced by DNA damage

A. Immunoblotting of HeLa and HeLa *CCNF K/O* after treatment with IR and recovery as indicated.
B. Immunoblotting after immunoprecipitation of GFP-cyclin F in HEK293T cells treated with IR for the indicated time (h= hour) (*left panel*) . Input samples before immunoprecipitation are presented in the *right panel*.
C. Immunoblotting after immunoprecipitation of GFP-cyclin F in HEK293T cells treated with or without Camptothecin (CPT) for the indicated time (h=hour)(*left panel*). Input samples before immunoprecipitation are presented in the *right panel*.
D. Immunoblotting after isolation of endogenous ubiquitinated proteins using recombinant GST-tagged UBA domain of UBQLN1 protein in HeLa or HeLa *CCNF K/O* treated with IR and/or MG132 as indicated. Input samples before immunoprecipitation are presented in the *right panel*.

Figure S4

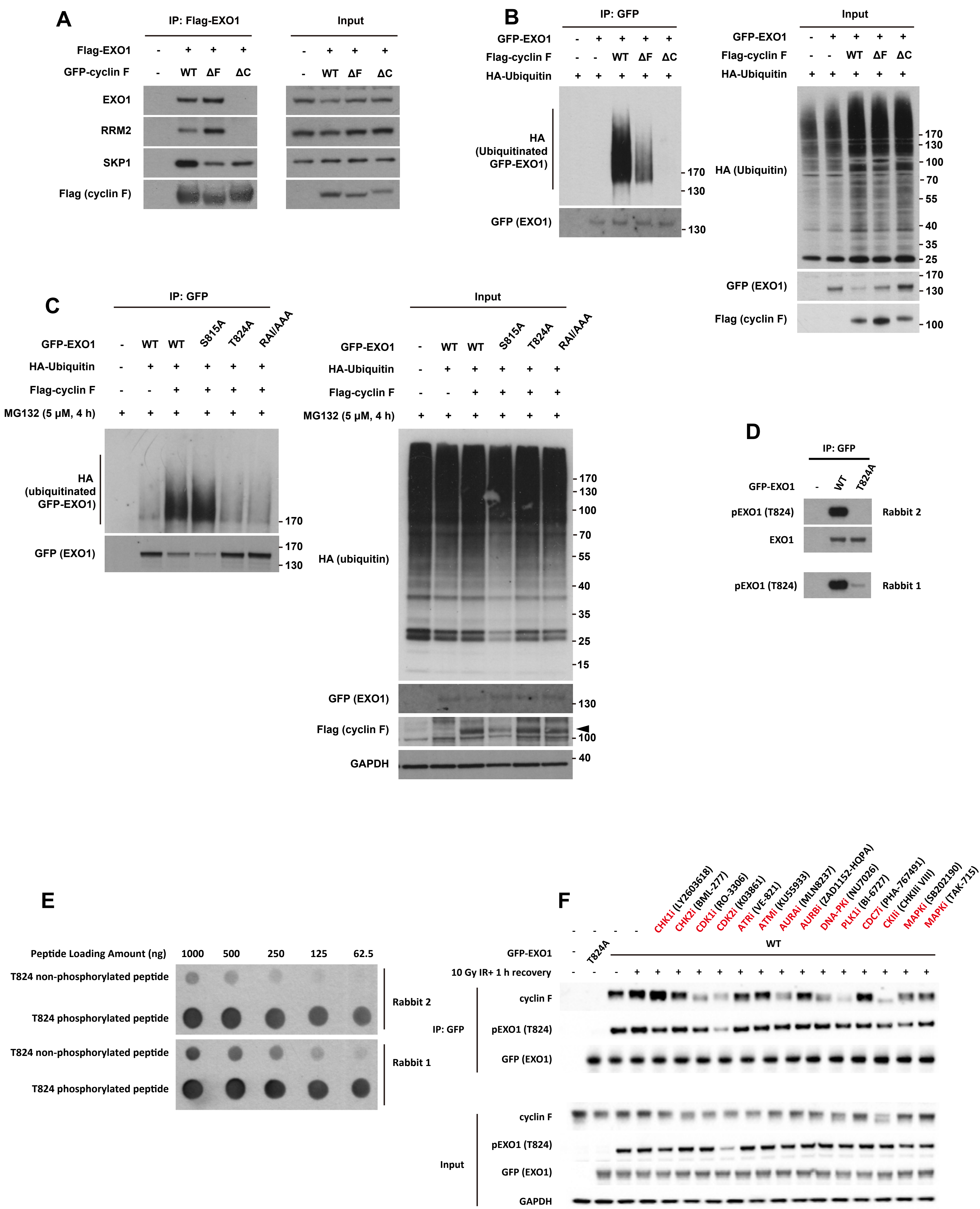

A
IP: Flag-EXO1
Input
Flag-EXO1
GFP-cyclin F
WT ΔF ΔC
EXO1
RRM2
SKP1
Flag (cyclin F)
B
IP: GFP
Input
GFP-EXO1
Flag-cyclin F
HA-Ubiquitin
HA (Ubiquitinated GFP-EXO1)
GFP (EXO1)
HA (Ubiquitin)
Flag (cyclin F)
C
IP: GFP
Input
GFP-EXO1
WT WT S815A T824A RAI/AAA
HA-Ubiquitin
Flag-cyclin F
MG132 (5 μM, 4 h)
HA (ubiquitinated GFP-EXO1)
GFP (EXO1)
HA (ubiquitin)
GAPDH
D
IP: GFP
GFP-EXO1
pEXO1 (T824)
EXO1
Rabbit 2
Rabbit 1
E
Peptide Loading Amount (ng)
1000 500 250 125 62.5
T824 non-phosphorylated peptide
T824 phosphorylated peptide
F
CHK1i (LY2603618)
CHK2i (BML-277)
CDK1i (RO-3306)
CDK2i (K03861)
ATRi (VE-821)
ATMi (KU55933)
AURAi (MLN8237)
AURBi (ZAD1152-HQPA)
DNA-PKi (NU7026)
PLK1i (BI-6727)
CDC7i (PHA-767491)
CKIIi (CHKII VIII)
MAPKi (SB202190)
MAPKi (TAK-715)
T824A
10 Gy IR+ 1 h recovery
cyclin F
pEXO1 (T824)
GFP (EXO1)
GAPDH

Figure S4. Phosphorylation of T824 in EXO1 is required for interaction with cyclin F

A. Immunoblotting after expression of GFP-cyclin F Wild Type (WT), GFP-cyclin F L35A/ P36A (ΔF, which disrupts the F box domain), GFP-cyclin F M309A (ΔC, which disrupts the cyclin domain) with Flag-EXO1, and immunoprecipitation (IP) of Flag-EXO1 isolated *via* Flag agarose beads. Input samples are presented in the right panel.
B. Immunoblotting after co-expression of Flag-cyclin F WT, Flag-cyclin F ΔF or Flag-cyclin F ΔC and GFP-EXO1 with HA-ubiquitin in HEK293T. GFP-EXO1 is isolated *via* GFP beads pulldown after denaturation. Input samples are presented in the *right panel*.
C. Immunoblotting after coexpression of GFP-EXO1 WT or indicated mutants with Flag-cyclin F and HA-ubiquitin in HEK293T, GFP-EXO1 was isolated via GFP beads pulldown after denaturation. Input samples are presented in the *right panel*.
D. Immunoblotting of custom- generated pT824 antibody using immunoprecipitated GFP-EXO1 WT or GFP-EXO1 T824A from HEK293T.
E. Different amounts of T824 phosphorylated or non-phosphorylated EXO1 peptides (in indicated quantities) were spotted on nitrocellulose membrane. After air-drying, the membrane is blocked with milk and immunoblotted for pT824 EXO1 using site-specific antibodies generated in two rabbits (1,2 as indicated).
F. Immunobloting of immunoprecipitated GFP-EXO1 from HEK293T cells after treatment with the indicated kinase inhibitors and IR.

Figure S5

A

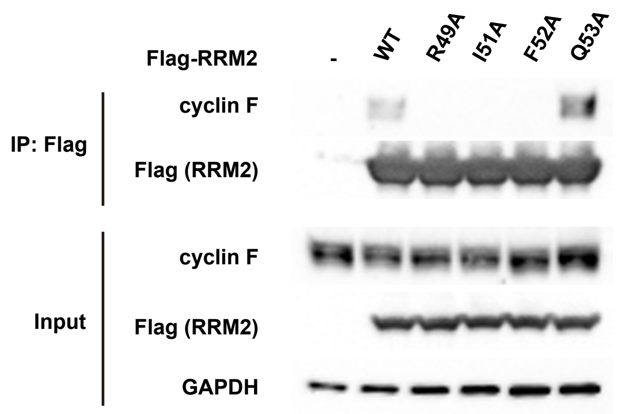

B

Protein ID	Pattern: TP.{10,25}R.IF
ACADL	...TPSAKKLTDIGIRRIF...
MIB1	...TPGSERVLLESRRKSKKITARGIFAGARV...
GALC	...TPDTGGVFIAGRVNKGGILIRSARGIFFWIFA...
XPP2	...TPSAFQKEAYTRVLIGNIDLSRLIFPAATS...
ATL4	...TPLGSPAAYWKRVGHSACSASCGKGVWRPIFLCISR...
COPB	...TPEKALSGYCGFMAANLYARSIFGEDAL...
LRC36	...TPLRTLLLSPGTSEHRKIFTKRSL...
RRM2	...TPPALSGTRVLASKTARRIFQEPTE...
RS4X	...TPEEAKYKLCKVRKIFVGTKG...
PNISR	...TPGPMDIVPPSEDSNSQDSGEFAPDNRHIFNQNNH...
NCBP1	...TPPPHTEDSVYPMPRVIF...
TRPM8	...TPNLVISVTGGAKNFALKPRMRKIF...
XRN1	...TPYYGCTGEVQDSGDVITEGRIRVIF...
BRWD3	...TPVDFSTVKETLEAGNYGSPLEFYKDVRQIF...
GALNS	...TPPSDRAIDGLNLLPTLLQGRLMDRPIF...
HPS1	...TPPMDALQIAEDTLQTLVPHCPVPSGPRRIF...
BRWD1	...TPMDFGTVRETLDAGNYDSPLEFCKDIRLIF...
CCNK	...TPSQLEGLDPATEARYRREGARFIF...
NR1D1	...TPPRSPSPEPTVEDVISQVARAHREIF...
TRI77	...TPNCCPVCREISQQMYFKRIIF...
NEK9	...TPTERPHGSDICTSWPRPIF..
PDS5A	...TPEQFQLCALVINDECYQVRQIF...
PHIP	...TPMDFATVRETLEAGNYESPMELCKDVRLIF...
ZN717	...TPGKSPMNVMNVENPFIRRQIF...
EXO1	...TPEAEEDIFNKPECGRVQRAIF...
TMG4	...TPGNLERECNEELCNYEEAREIF...
KKCC2	...TPAFMAPESLSETRKIF...
PRC1	...TPGKARKLNTTTMSNATANSSIRPIF...
NARR	...TPRPQLAAEESPRPRVIF...
PAX4	...TPHSGSETPRGTHPGTGHRNRTIF...
ENTP8	...TPTFLGATAGMRLLSRKNSSQARDIF...
OR6N2	...TPMVNPIIYSLRNKEIIKAIKRTIF...
TR64C	...TPCYISLSEDVRRVIF...
NMD3A	...TPTVVMFGCDMESIRRIF...

C

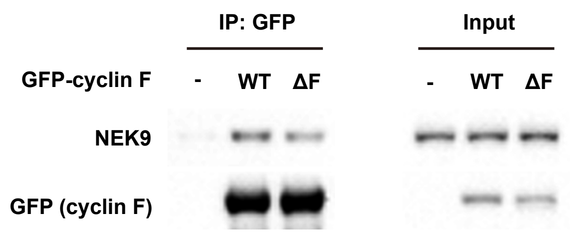

D

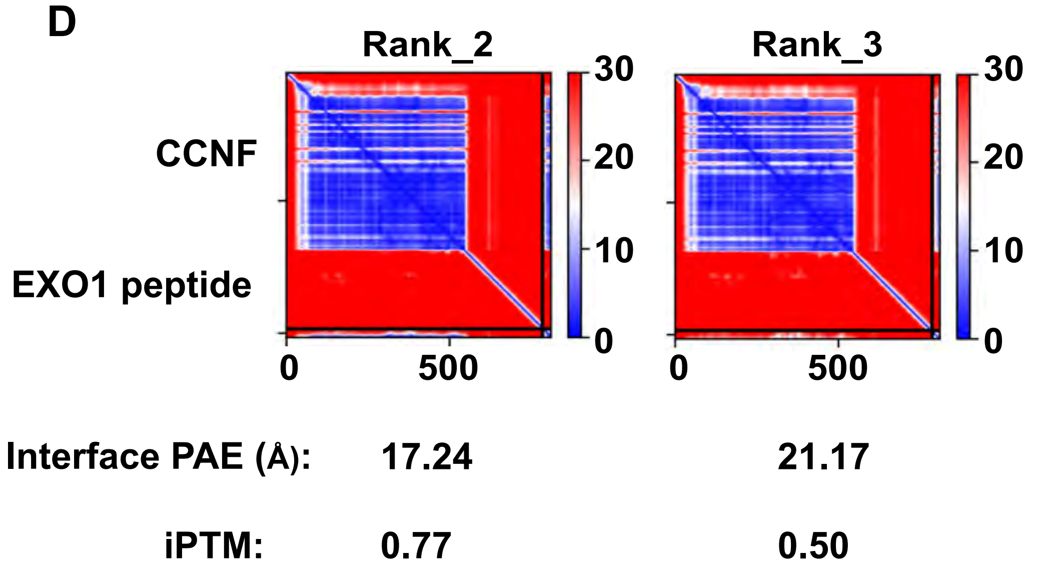

Figure S5. Identification of potential cyclin F substrates through the F-deg

A. Immunoblotting of immunoprecipitated Flag-RRM2 WT, Flag-RRM2 R49A, Flag-RRM2 I51A, Flag-RRM2 F52A, Flag-RRM2 Q53A from HEK293T. Input is presented.
B. Result of proteome-wide search of the F-deg *via* Scansite 4.0 using TP.{10,25}R.IF as the regular expression pattern.
C. Immunobloting after immunoprecipitation of GFP-Cyclin F WT or GFP-Cyclin F ΔF from HEK293T.
D. PAE value plots for second and third-ranked prediction from AlphaFold multimer.

Figure S6

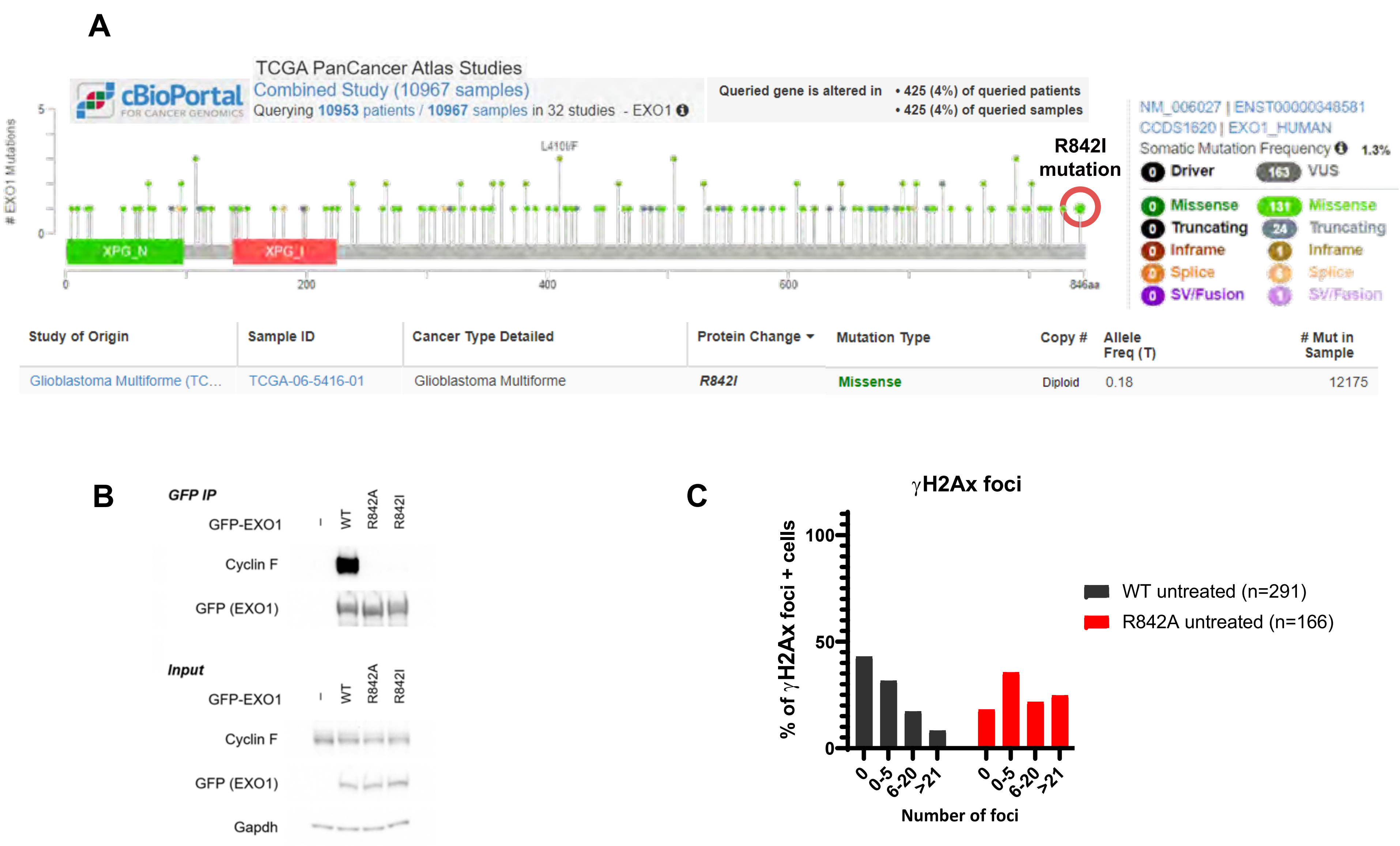
A
TCGA PanCancer Atlas Studies
cBioPortal
FOR CANCER GENOMICS
Combined Study (10967 samples)
Querying 10953 patients / 10967 samples in 32 studies - EXO1
Queried gene is altered in
• 425 (4%) of queried patients
• 425 (4%) of queried samples
EXO1 Mutations
L410I/F
R842I
mutation
XPG_N
XPG_I
0
200
400
600
846aa
NM_006027 | ENST00000348581
CCDS1620 | EXO1_HUMAN
Somatic Mutation Frequency 1.3%
0 Driver
163 VUS
0 Missense
131 Missense
0 Truncating
24 Truncating
0 Inframe
Inframe
0 Splice
Splice
0 SV/Fusion
SV/Fusion
Study of Origin
Sample ID
Cancer Type Detailed
Protein Change
Mutation Type
Copy #
Allele Freq (T)
Mut in Sample
Glioblastoma Multiforme (TC...
TCGA-06-5416-01
Glioblastoma Multiforme
R842I
Missense
Diploid
0.18
12175
B
GFP IP
GFP-EXO1
-
WT
R842A
R842I
Cyclin F
GFP (EXO1)
Input
GFP-EXO1
-
WT
R842A
R842I
Cyclin F
GFP (EXO1)
Gapdh
C
γH2Ax foci
% of γH2Ax foci + cells
100
50
0
WT untreated (n=291)
R842A untreated (n=166)
0
0-5
6-20
>21
0
0-5
6-20
>21
Number of foci

Figure S6. Mutation in the EXO1 degron (R842I) in glioblastoma

A. Snapshot of EXO1 mutations identified in the cBioportal encompassing 10932 samples in 32 studies. R842I mutation identified in glioblastoma multiforme within the RxIF motif is highlighted.
B. Immunoblotting of indicated proteins after immunoprecipitation of GFP-EXO1 WT, GFP-EXO1 R842A, GFP-EXO1 R842I. Input samples before immunoprecipitation are presented in the *bottom panel*.
C. Quantification of γH2Ax foci in LN229 stable cells expressing HA-EXO1 WT or HA-EXO1 R842A after 10 Gy IR treatment.

Figure S7

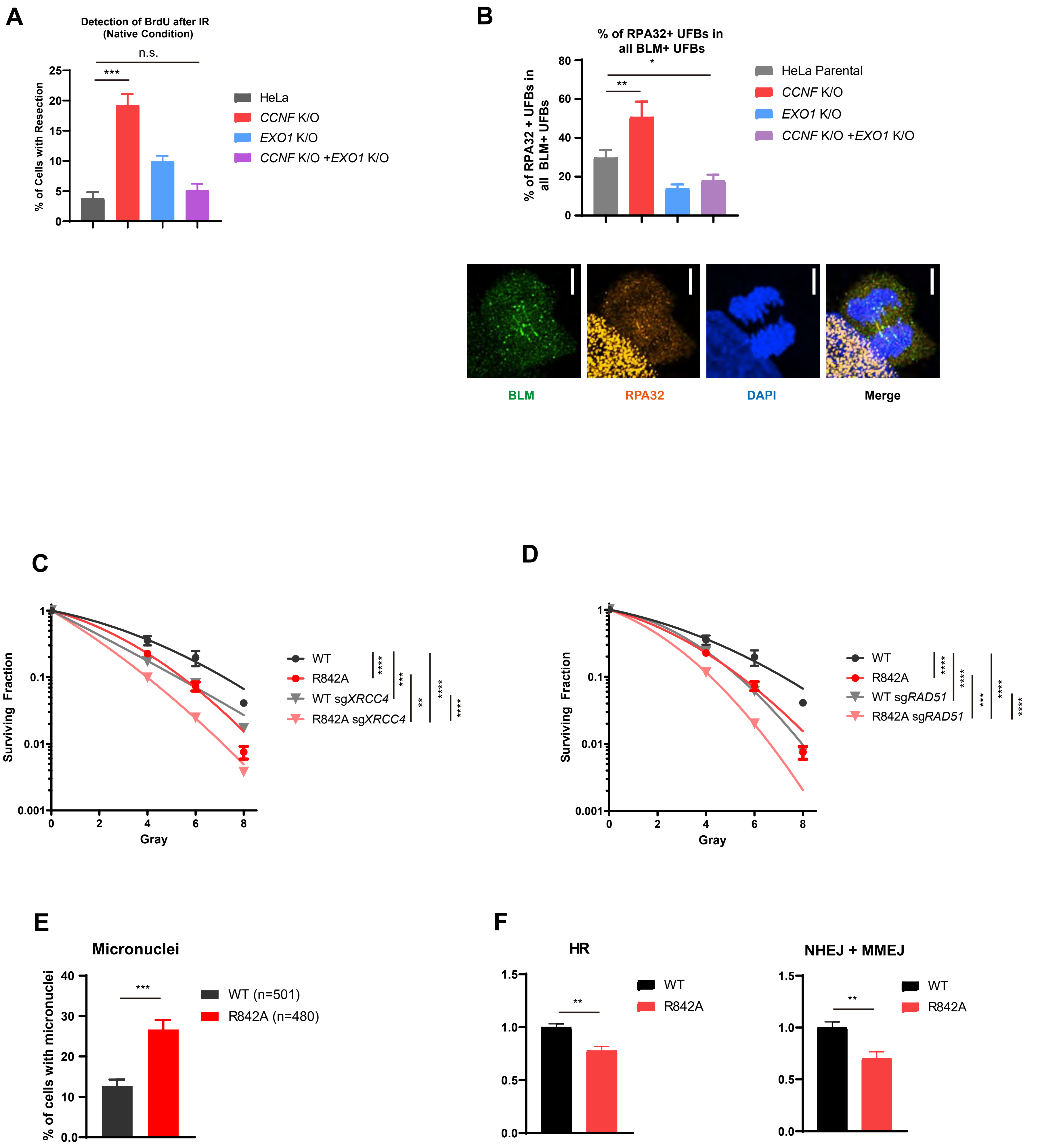
A
Detection of BrdU after IR
(Native Condition)
n.s.
***
% of Cells with Resection
HeLa
CCNF K/O
EXO1 K/O
CCNF K/O +EXO1 K/O
B
% of RPA32+ UFBs in
all BLM+ UFBs
*
**
% of RPA32 + UFBs in
all BLM+ UFBs
HeLa Parental
CCNF K/O
EXO1 K/O
CCNF K/O +EXO1 K/O
BLM
RPA32
DAPI
Merge
C
Surviving Fraction
Gray
WT
R842A
WT sgXRCC4
R842A sgXRCC4
D
Surviving Fraction
Gray
WT
R842A
WT sgRAD51
R842A sgRAD51
E
Micronuclei
% of cells with micronuclei
***
WT (n=501)
R842A (n=480)
F
HR
NHEJ + MMEJ
**
WT
R842A

Figure S7. ***CCNF*** **K/O or EXO1 R842A expression lead to genome instability**

A. Quantification of BrdU signal in HeLa parental cells, HeLa *CCNF K/O* , HeLa *EXO1 K/O* and HeLa *CCNF K/O EXO1 K/O* .Cells were seeded at 50% density and labelled with 10 M BrdU for 24 hours before being treated with 10 Gy IR and allowed to recover for 3 hours. Error bars represent standard deviations of three biological replicates. Statistical analysis was performed using two-tailed unpaired t-test. *** indicates P ≤ 0.001, n.s. = not significant.
B. Quantification of RPA32 positive anaphase ultra-fine bridges (UFBs) in HeLa parental cells, HeLa *CCNF K/O* , HeLa *EXO1 K/O* and HeLa *CCNF K/O EXO1 K/O* . Cells were treated with 10 Gy IR and allowed to recover for 12 hours before UFB detection. At least 50 events were quantified for each condition. Error bars represent standard deviation of three biological replicates. Statistical analysis was performed using two-tailed unpaired t-test. * indicates P ≤ 0.05, ** indicates P ≤ 0.01, n.s . indicates not significant. Representative images are provided. Scale bar = 5 µm.
C. LN229 stable cells, expressing HA-EXO1 WT or HA-EXO1 R842A, were transfected with a control sgRNA or sgRNA targeting XRCC4 as indicated. Cells seeded for colony formation assay and challenged with the indicated doses of IR. Error bars represent standard deviations of three biological replicates.
D. LN229 stable cells indicated in C were transfected with a control sgRNA or sgRNA targeting RAD51 as indicated. Cells were seeded for colony formation assay and challenged with the indicated doses of IR. Error bars represent standard deviations of three biological replicates.
E. Quantification of micronuclei in LN229 expressing HA-EXO1 WT or HA-EXO1 R842A. Error bars indicate standard deviations of three biological replicates. n represents total events quantified in all three replicates. Statistical analysis was performed using two-tailed unpaired t-test. *** indicates P ≤ 0.001.
F. Quantification of events using HR and MMEJ+NHEJ reporter assay. Error bars represents standard deviation of three biological replicates. Repair efficiency was normalized against that of the cells expressing control sgRNA. Statistical analysis was performed using two-tailed unpaired t-test. ** indicates P ≤ 0.01.

Table S1: *z-ratio* scores from the CRISPR screen to identify genes controlling cell proliferation in LN229. (essential genes and control essential genes are highlighted).

Table S2: *z-ratio* scores from the CRISPR screen to identify genes modulating sensitivity to IR in LN229.

Table S3: Cyclin F interactome by TurboBioID/ Mass Spectrometry. Free- label quantification of three biological replicates from three samples: 1.no biotin; 2. + biotin; 3. + biotin + MLN4924

Table S4: Proteomics after Flag immunoprecipitation of GFP-EXO1 WT and GFP-EXO1 T824A. Presented in Figure 4E.
